# Supplementary material for: Fission Yeast NDR/LATS Kinase Orb6 Regulates Exocytosis via Phosphorylation of the Exocyst Complex
Source: Cell Rep. 2019 Feb 5;26(6):1654–1667.e7. doi: 10.1016/j.celrep.2019.01.027 (PMC6367570; doi:10.1016/j.celrep.2019.01.027)
Supplement: Document S1. Figures S1–S5 [file mmc1.pdf]

Cell Reports, Volume *26*

Supplemental Information

Fission Yeast NDR/LATS Kinase Orb6 Regulates Exocytosis via Phosphorylation of the Exocyst Complex

Ye Dee Tay, Marcin Leda, Christos Spanos, Juri Rappsilber, Andrew B. Goryachev, and Kenneth E. Sawin

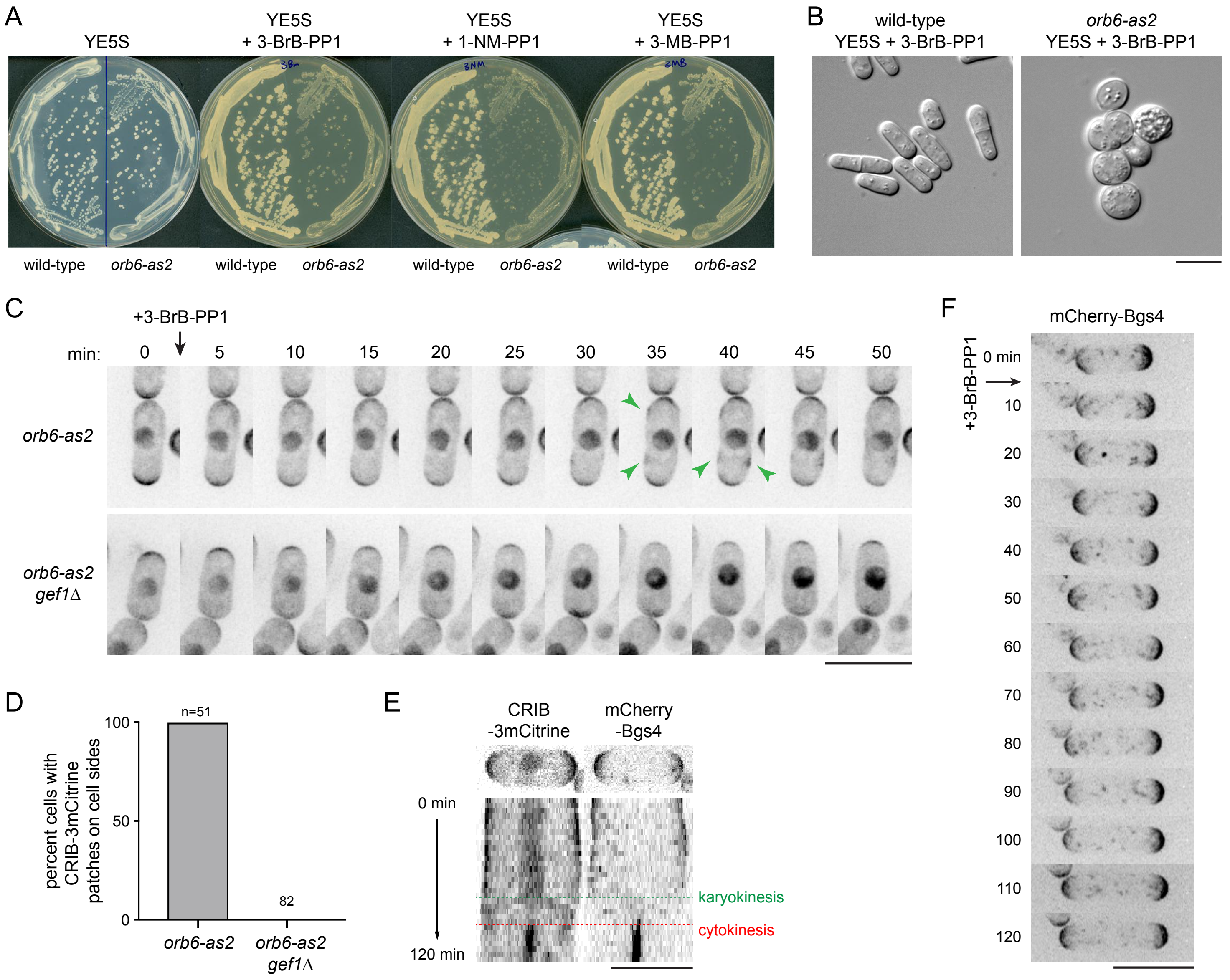

Figure S1. Additional characterization of *orb6-as2* and effects of 3-BrB-PP1. Related to Figures 1 and 2.
(A) Wild-type and *orb6-as2* colonies after replica-plating to the different indicated nucleotide-competitive analogs (each analog at 30 μM final concentration). All analogs inhibited growth of *orb6-as2* cells. Plates were incubated at 32ºC for 2 days.
(B) DIC images of wild-type and *orb6-as2* cells taken from YE5S + 3-BrB-PP1 plate. On solid media at 32°C, Orb6-inhibited cells are rounder than in liquid medium at 25°C; this is partly due to elevated temperature (see Figure 1).
(C) CRIB-3mCitrine (Cdc42-GTP reporter) localization in interphase *orb6-as2* and *orb6-as2 gef1*Δ cells before and after Orb6 inhibition with 3-BrB-PP1. After Orb6 inhibition, ectopic patches of CRIB-3mCitrine are observed in *orb6-as2* cells (arrowheads) but not in *orb6-as2 gef1*Δ cells.
(D) Quantification of ectopic CRIB-3mCitrine patches from experiments as in (C).
(E) Temporal correlation of CRIB-3mCitrine and mCherry-Bgs4 localization to cell tips during interphase and to the division site during cell division.
(F) Addition of 3-BrB-PP1 (30 μM) to wild-type cells does not affect mCherry-Bgs4 localization or interphase cell elongation.
Bars, 10 μm. Three biological replicates were performed for (C) and (E). Experiments for (A), (B) and (F) were performed once.

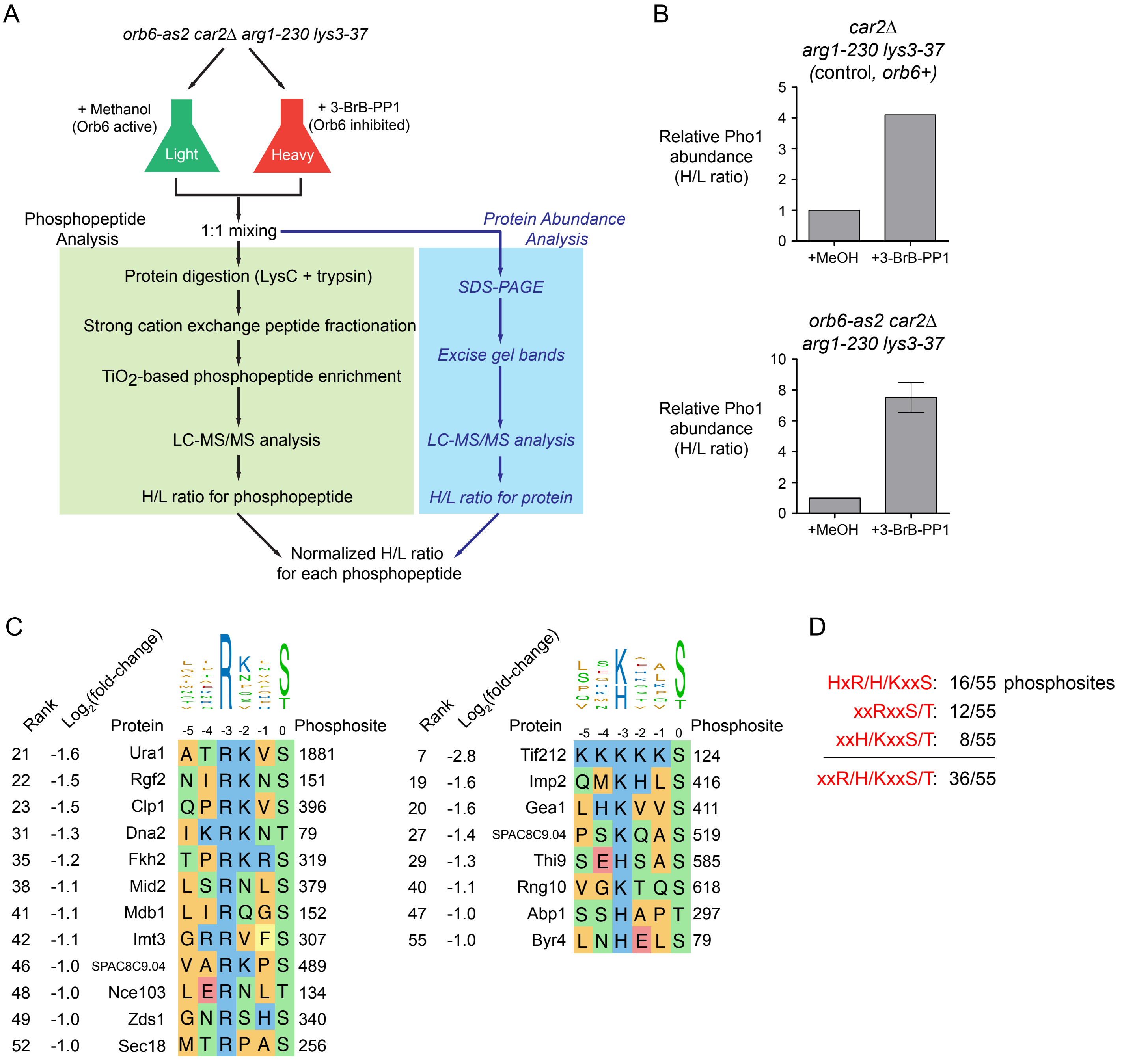

Figure S2. Quantitative global phosphoproteomics analysis after Orb6 inhibition. Related to Figure 4.
(A) Workflow for SILAC quantitative global phosphoproteomics analysis. Because relative abundance of individual proteins may change as a result of Orb6 inhibition, abundance of individual proteins before and after Orb6 inhibition is used to normalize H/L ratios of the relevant phosphopeptides.
(B) Relative abundance of acid phosphatase Pho1 after 3-BrB-PP1 addition to control wild-type (*orb6+*) cells and *orb6-as2* cells. Error bar indicates SD from three biological replicates. Relative abundances of other acid phosphatases SPBC4.06 and Pho4 were not significantly changed after 3-BrB-PP1 addition.
(C) Alignment of selected highest-confidence Orb6-dependent phosphosites containing basic residues R or H/K at -3 position. All phosphosites shown were quantified in all three replicate global phosphoproteomics experiments and showed a two-fold or greater mean decreased phosphorylation after Orb6 inhibition. Phosphosites are ranked by fold-decrease in phosphorylation (see Figure 4B and Table S2).
(D) Consensus summary of features of highest-confidence Orb6-dependent phosphosites from (C), together with sites with perfect match to NDR/LATS consensus HxR/H/KxxS/T (Figure 4B). 36 out of 55 sites include a basic residue at -3 position.

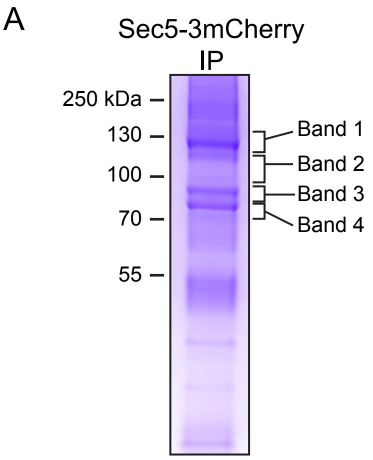

B

Protein	H/L Ratio	Peptide Counts
Sec5	1.05	51
Sec3	1.17	56
Sec8	0.99	89
Sec6	0.97	54
Sec10	0.79	14
Sec15	0.94	18
Exo84	0.83	10
Exo70	0.96	10

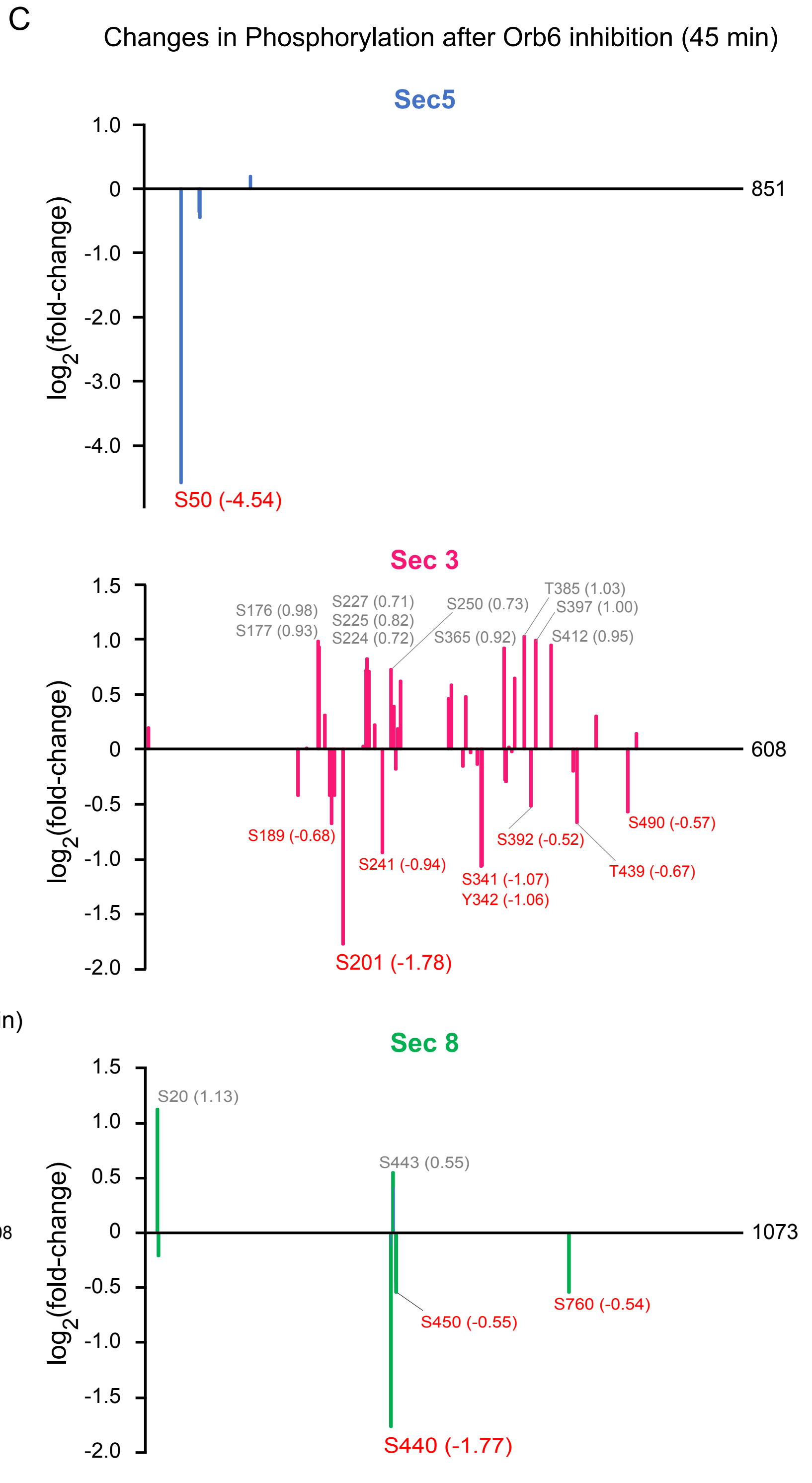

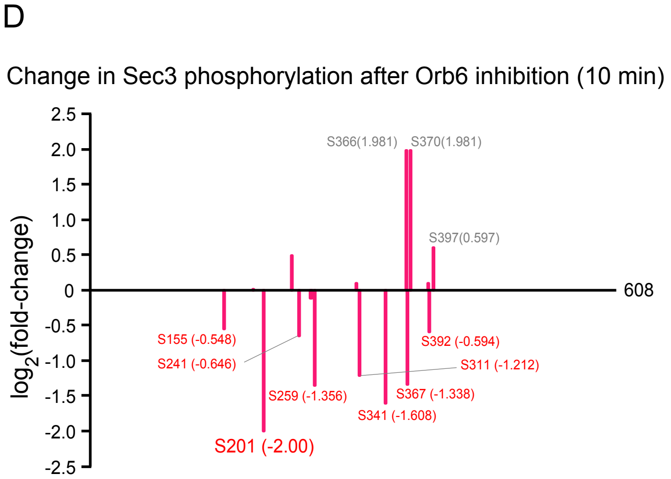

Figure S3. Additional confirmation of changes in phosphorylation of exocyst proteins after Orb6 inhibition. Related to Figure 4.
(A) SDS-PAGE showing immunoprecipitated Sec5-3mCherry and coimmunoprecipitated proteins, from a mixture of SILAC "heavy"-labelled Orb6-inhibited *orb6-as2* cells and "light"-labelled non-inhibited *orb6-as2* cells. Heavy-labelled cells were treated with 3-BrB-PP1, while light-labelled cells were treated with methanol. Indicated bands were excised and processed for tandem mass spectrometry.
(B) Relative abundances of exocyst proteins present in SILAC Sec5-3mCherry immunoprecipitate from (A) before and after Orb6 inhibition, based on SILAC heavy:light ratio (H/L ratio). All ratios are close to 1, suggesting that Orb6 inhibition does not grossly alter exocyst integrity. Note that H/L ratios here are specific to individual proteins immunoprecipitating with Sec5-3mCherry before vs. after Orb6 inhibition and are not indicating protein stoichiometry within the exocyst complex.
(C) Fold-changes in phosphorylation of individual phosphosites in Sec5, Sec3, and Sec8 after Orb6 inhibition for 45 minutes. X-axes show position of phosphosites in the respective proteins (Sec5 is 851 amino acids, Sec3 is 608 amino acids, and Sec8 is 1073 amino acids). Y-axes show $\log_2$(fold-change) in phosphorylation of relevant phosphosites after Orb6 inhibition. Among exocyst proteins, only Sec5, Sec3 and Sec8 showed significant changes in phosphorylation after Orb6 inhibition.
(D) Fold-changes in Sec3 phosphorylation after Orb6 inhibition for 10 minutes. In the 10-minute inhibition experiment, phosphorylation of Sec5-S50 and Sec8-440 was not detectable.

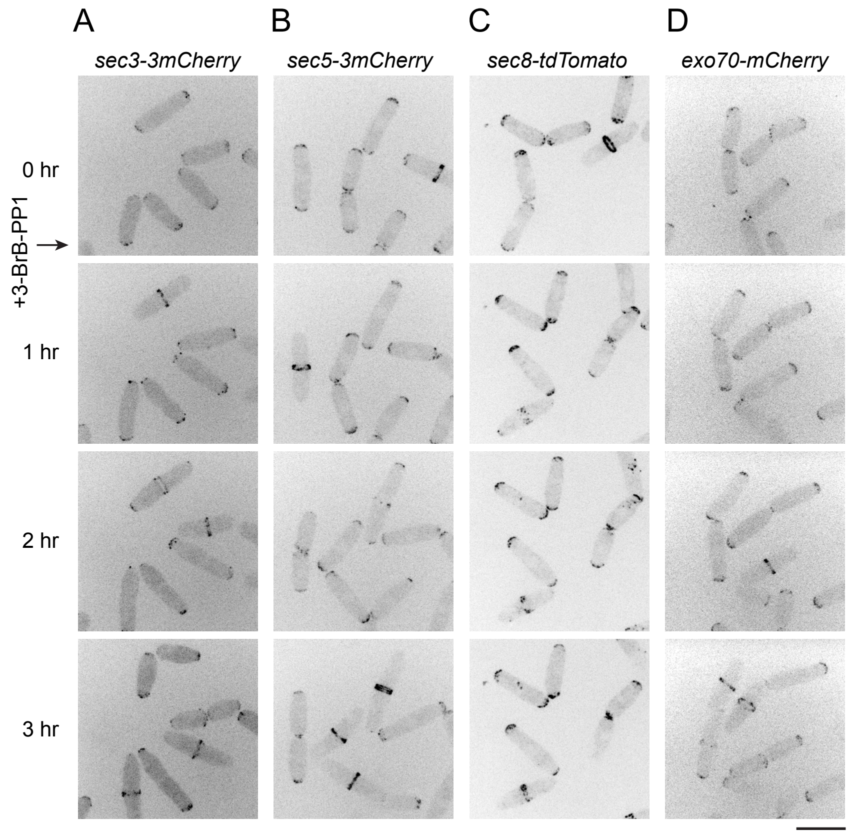

Figure S4. 3-BrB-PP1 treatment of wild-type cells does not affect exocyst localization. Related to Figure 6.
Localization of the indicated fluorescent-tagged exocyst proteins in wild-type (*orb6+*) cells, before 3-BrB-PP1 addition and at different times after 3-BrB-PP1 addition. In each column (A-D), the same fields of cells were imaged at all timepoints.
Bar, 10 μm. Imaging experiments for (A-D) were performed once.

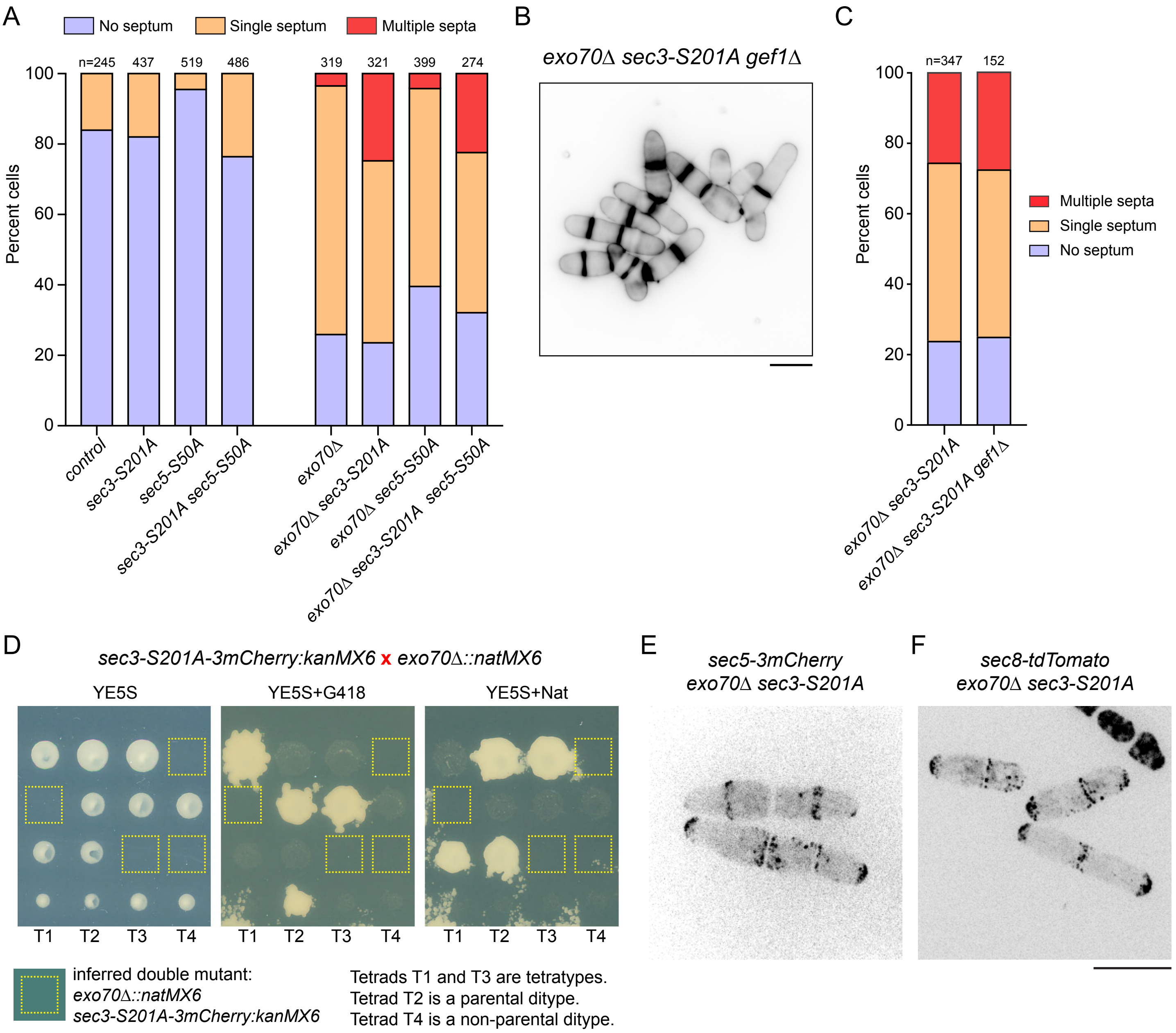

Figure S5. Further characterization of *sec3-S201A* and *sec5-S50A* phenotypes. Related to Figure 7.
(A) Septation index in cells of the indicated genotypes, grown at 36°C. See also Figure 7D
(B) Calcofluor staining of *exo70Δ sec3-S201A gef1Δ* cells.
(C) Septation index in cells of the indicated genotypes (25°C). Data in left-hand column are reproduced from Figure 7D.
(D) *sec3-S201A-3mCherry* is synthetically lethal with *exo70Δ*. As a result, Sec3-S201A-3mCherry localization in *exo70Δ* background cannot be investigated.
(E and F**)** Localization of Sec5-3mCherry (E) and Sec8-tdTomato (F) to cell tips and the septation zone in *exo70Δ sec3-S201A* backgrounds. In both strains, joined sister cells indicate cell-separation defects (see also Figure 7).
Bars, 10 µm. Imaging experiments and genetic cross for (A-F) were performed once.
