# Supplementary material for: Fission Yeast NDR/LATS Kinase Orb6 Regulates Exocytosis via Phosphorylation of the Exocyst Complex
Source: Cell Rep. 2019 Feb 5;26(6):1654–1667.e7. doi: 10.1016/j.celrep.2019.01.027 (PMC6367570; doi:10.1016/j.celrep.2019.01.027)
Supplement: Document S2. Article plus Supplemental Information [file mmc13.pdf]

Cell Reports

Article

Fission Yeast NDR/LATS Kinase Orb6 Regulates Exocytosis via Phosphorylation of the Exocyst Complex

Graphical Abstract

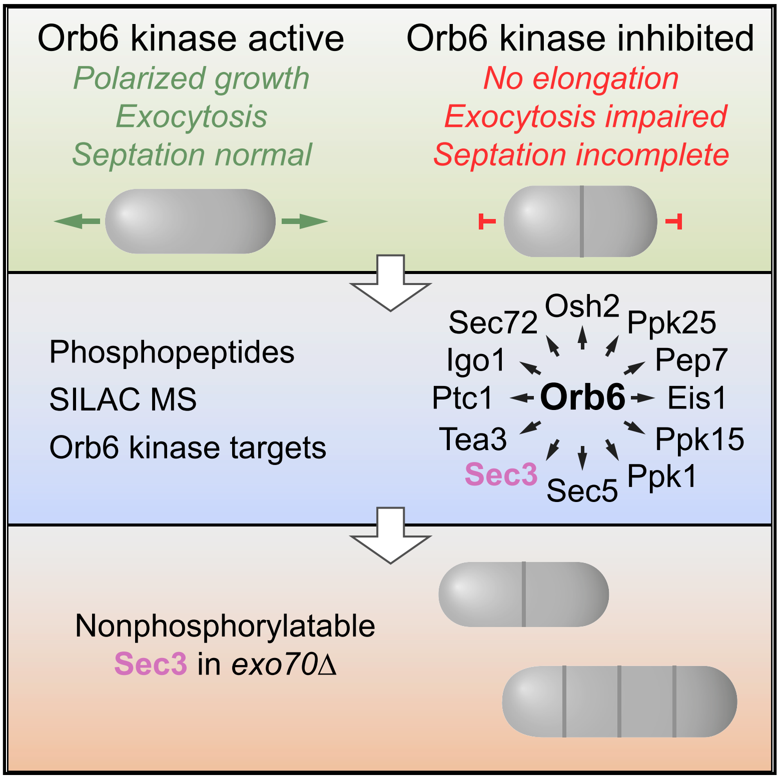

Highlights

- Inhibiting Orb6 leads to cessation of cell elongation and impaired exocytosis
- These and related phenotypes are independent of the Cdc42 GEF Gef1
- Phosphoproteomics reveals multiple Orb6 targets involved in membrane trafficking
- Exocyst protein Sec3 is an important Orb6 substrate for daughter-cell separation

Authors

Ye Dee Tay, Marcin Leda, Christos Spanos, Juri Rappsilber, Andrew B. Goryachev, Kenneth E. Sawin

Correspondence

andrew.goryachev@ed.ac.uk (A.B.G.), ken.sawin@ed.ac.uk (K.E.S.)

In Brief

NDR/LATS kinases are known primarily for their role in controlling cell and tissue proliferation and morphogenesis, e.g., via regulation of transcription in the Hippo pathway. Using fission yeast *S. pombe* as a model system, Tay et al. show that the NDR/LATS kinase Orb6 is a major regulator of exocytosis.

Tay et al., 2019, Cell Reports *26*, 1654–1667
February 5, 2019 © 2019 The Authors.
https://doi.org/10.1016/j.celrep.2019.01.027

OPEN ACCESS
CellPress

Cell Reports
Article

Fission Yeast NDR/LATS Kinase Orb6 Regulates Exocytosis via Phosphorylation of the Exocyst Complex

Ye Dee Tay,[1,4] Marcin Leda,[2,4] Christos Spanos,[1] Juri Rappsilber,[1,3] Andrew B. Goryachev,[2,*] and Kenneth E. Sawin[1,5,*]
[1]Wellcome Centre for Cell Biology, School of Biological Sciences, University of Edinburgh, Michael Swann Building, Max Born Crescent, Edinburgh EH9 3BF, UK
[2]SynthSys-Centre for Synthetic and Systems Biology, School of Biological Sciences, University of Edinburgh, CH Waddington Building, Max Born Crescent, Edinburgh EH9 3BF, UK
[3]Chair of Bioanalytics, Institute of Biotechnology, Technische Universität Berlin, Berlin, 13355, Germany
[4]These authors contributed equally
[5]Lead Contact
*Correspondence: andrew.goryachev@ed.ac.uk (A.B.G.), ken.sawin@ed.ac.uk (K.E.S.)
https://doi.org/10.1016/j.celrep.2019.01.027

SUMMARY

NDR/LATS kinases regulate multiple aspects of cell polarity and morphogenesis from yeast to mammals. Fission yeast NDR/LATS kinase Orb6 has been proposed to control cell polarity by regulating the Cdc42 guanine nucleotide exchange factor Gef1. Here, we show that Orb6 regulates polarity largely independently of Gef1 and that Orb6 positively regulates exocytosis. Through Orb6 inhibition *in vivo* and quantitative global phosphoproteomics, we identify Orb6 targets, including proteins involved in membrane trafficking. We confirm Sec3 and Sec5, conserved components of the exocyst complex, as substrates of Orb6 both *in vivo* and *in vitro*, and we show that Orb6 kinase activity is important for exocyst localization to cell tips and for exocyst activity during septum dissolution after cytokinesis. We further find that Orb6 phosphorylation of Sec3 contributes to exocyst function in concert with exocyst protein Exo70. We propose that Orb6 contributes to polarized growth by regulating membrane trafficking at multiple levels.

INTRODUCTION

The ability to establish, maintain, and alter polarity is central to the function of nearly all eukaryotic cell types (Campanale et al., 2017; Mayor and Etienne-Manneville, 2016; Rodriguez-Boulan and Macara, 2014; Schelski and Bradke, 2017; St Johnston and Ahringer, 2010). Dysregulation of cell polarity is associated with multiple pathologies, including tumorigenesis and neurodegenerative disease (Martin-Belmonte and Perez-Moreno, 2011; Millecamps and Julien, 2013). NDR/LATS kinases are members of a subfamily of the AGC serine-threonine kinases and are important for polarized cellular differentiation in multiple systems (Hergovich et al., 2006). NDR/LATS kinases such as Trc (*Drosophila*), NDR1 and NDR2 (NDR1/2) (mammals), and SAX-1/SAX-2 (*C. elegans*) share evolutionarily conserved functions in coordinating neurite branching and patterning of neuronal fields (Hergovich, 2016). Other NDR/LATS kinases such as Wts (*Drosophila*), LATS1 and LATS2 (LATS1/2) (mammals) and WTS-1 (*C. elegans*) regulate polarized differentiation of epithelia and other cell types (Furth and Aylon, 2017). In budding yeast *Saccharomyces cerevisiae* and fission yeast *Schizosaccharomyces pombe*, NDR/LATS kinases Cbk1 and Orb6, respectively, are required for polarized cellular morphogenesis (Bidlingmaier et al., 2001; Racki et al., 2000; Verde et al., 1998). Other essential biological processes regulated by NDR/LATS kinases include centrosome duplication, cell-cycle progression, autophagy, and apoptosis (Hergovich, 2016).

Identification of targets of NDR/LATS kinases is crucial for understanding how they regulate complex biological processes. To date, surprisingly few targets of NDR/LATS kinases are known (Hergovich, 2013). The best characterized targets in mammals and *Drosophila* are the transcriptional co-activators YAP and TAZ (targets of LATS1/2) and Yki (target of Wts), respectively (Yu and Guan, 2013). Phosphorylation of YAP, TAZ, and Yki is an important element of the Hippo pathway, a tumor suppressor pathway regulating cell shape and proliferation (Hansen et al., 2015). In addition, NDR1/2 phosphorylate p21 cyclin-dependent kinase inhibitor and MYPT1 phosphatase, which regulate the G1/S transition and G2 DNA damage checkpoint, respectively (Chiyoda et al., 2012; Cornils et al., 2011). In neurons, NDR1/2 phosphorylate AP2-associated kinase 1 (AAK1) and Rabin8, the guanine nucleotide exchange factor (GEF) for Rab8 guanosine triphosphatase (GTPase), which are involved in vesicle trafficking and are important for dendrite growth regulation and dendritic spine development, respectively (Ultanir et al., 2012).

In budding yeast, Cbk1 inactivation or inhibition affects both cell morphogenesis and asymmetry of gene expression between mother and daughter cell. Cbk1 phosphorylates the transcription factor Ace2 and the RNA-binding protein Ssd1, a translational regulator (Weiss, 2012). Cbk1 is also reported to phosphorylate Sec2, a GEF for the Rab GTPase Sec4 (Kurischko et al., 2008). In fission yeast, temperature-sensitive *orb6-25* mutants lose polarity at non-permissive temperature, and cells become round rather than rod shaped (Verde et al., 1998). *In vitro*, Orb6 can

This is an open access article under the CC BY license (http://creativecommons.org/licenses/by/4.0/).

phosphorylate the Ssd1 homolog Sts5 (Nuñez et al., 2016), as well as Gef1, a GEF for the Rho-family cell-polarity GTPase Cdc42 (Das et al., 2015). Phosphorylation of Gef1 serine-112 is thought to promote Gef1 association with 14-3-3 protein Rad24, restricting Gef1's ability to activate Cdc42 (Das et al., 2015). Accordingly, Orb6 inactivation leads to ectopic localization of active Cdc42 (Cdc42-GTP) on cell sides, with subsequent recruitment of formin For3 to these ectopic sites (Das et al., 2009). These events have been proposed to drive reorganization of actin cable nucleation and redirection of intracellular transport, leading to increased cell width in Orb6-inhibited cells (Das et al., 2009).

Here we show, in contrast to previous work, that several phenotypes associated with Orb6 inactivation, including increased cell width, are independent of Gef1. We further find that Orb6 positively regulates exocytosis, also independently of Gef1. To identify novel targets of Orb6, we performed quantitative global phosphoproteomics analyses of cells with decreased Orb6 kinase activity and generated a high-quality dataset of Orb6 targets *in vivo*. Targets include proteins involved in kinase signaling, membrane trafficking, and the exocyst complex, a conserved octameric complex involved in late stages of exocytosis (Kee et al., 1997; TerBush and Novick, 1995). The exocyst, which contains proteins that bind to secretory vesicles, as well as proteins that bind to the plasma membrane, mediates secretory vesicle docking to the plasma membrane before SNARE-mediated vesicle fusion (Heider and Munson, 2012; Wu and Guo, 2015). We show that Orb6 phosphorylates exocyst proteins *in vitro* and that exocyst phosphorylation *in vivo* mediates a subset of Orb6-dependent phenotypes. Overall, our results suggest that Orb6 regulates cell polarity via multiple targets involved in membrane trafficking and exocytosis.

RESULTS

Several Cell Growth and/or Polarity Phenotypes after Orb6 Inhibition *In Vivo* Are Gef1 Independent

Protein kinase function *in vivo* can be studied by mutating gatekeeper residues in the kinase ATP-binding pocket, making kinase activity sensitive to cell-permeable, nucleotide-competitive analogs (Bishop et al., 2000). An analog-sensitive *orb6* allele driven by the high-strength *nmt1* promoter has been described previously (Das et al., 2009). To investigate Orb6 function at native expression levels, we introduced the same mutation (M170A) at the endogenous *orb6* locus; throughout this work, we refer to our new allele as *orb6-as2.* We inhibited Orb6-as2 using the nucleotide competitive analog 3-BrB-PP1, although other analogs were equally effective (Figures S1A and S1B). For simplicity, we will refer to *orb6-as2* cells treated with 3-BrB-PP1 as Orb6-inhibited cells. Consistent with previous work (Das et al., 2009; Nuñez et al., 2016), Orb6 inhibition *in vivo* led to an increase in cell width, partial ectopic localization of Cdc42-GTP (imaged with CRIB-3mCitrine) (Mutavchiev et al., 2016) and the actin cytoskeleton away from cell tips (i.e., on cell sides), and cell-separation defects during septation (Figures 1A, 1C, S1C, and S1D; Video S1). These phenotypes were specific to *orb6-as2* cells treated with 3-BrB-PP1 (Figures 1C and S1B; Videos S2 and S3). Also consistent with previous work, ectopic localization of Cdc42-GTP and actin in Orb6-inhibited cells was suppressed by *gef1Δ* (Figures 1A, S1C, and S1D; Video S1) (Das et al., 2009).

However, in apparent contrast to the same previous work (Das et al., 2009), we found that increased cell width in Orb6-inhibited cells was not suppressed by *gef1Δ* (Figures 1A and 1C; Video S1). Because this work had investigated *gef1Δ* suppression of cell-width increase only in temperature-sensitive *orb6-25* mutants, not in Orb6-inhibited cells, we also examined *orb6-25* cells. The width increase of *orb6-25* cells at 37°C (restrictive temperature) was greater than that of Orb6-inhibited cells at 25°C, with many *orb6-25* cells becoming round (Figures 1B, 1C, and S1B) (Verde et al., 1998). We found that this difference can be attributed to the high temperature required to reveal the *orb6-25* phenotype, because Orb6 inhibition by 3-BrB-PP1 at 37°C also led to a greater width increase (Figure 1C). We suggest that the original *orb6-25* phenotype (Verde et al., 1998) is a synthetic phenotype that depends on both temperature-dependent inactivation of *orb6* function and increased temperature. In any case, similar to Orb6-inhibited cells, we found that width increase in *orb6-25* cells was not suppressed by *gef1Δ*, although a small proportion (~15%) of *orb6-25 gef1Δ* cells at restrictive temperature displayed widths within the range of *orb6-25* and *orb6-25 gef1Δ* cells at permissive temperature (Figures 1B and 1C).

Time-lapse observation of Cdc42-GTP localization revealed that Orb6-inhibited cells almost completely ceased to elongate, even when they remained largely polarized (based on Cdc42-GTP retention at cell tips) (Figures 1D and 1E; Video S4). This was surprising, because Cdc42-GTP enrichment on the plasma membrane is normally closely correlated with active cell growth (Figure S1E) (Tatebe et al., 2008). Cessation of elongation also occurred in Orb6-inhibited *gef1Δ* cells (Figures 1D–1G; Video S4), which do not show ectopic Cdc42-GTP localization. Further analysis indicated that cell-volume expansion over time is significantly decreased in Orb6-inhibited cells (both wild-type and *gef1Δ*) (Figures 1E–1G) (see STAR Methods); that is, cell widening in Orb6-inhibited cells does not compensate for cessation of cell elongation.

We conclude that in addition to increased cell width and impaired cell separation, a key phenotype associated with Orb6 inhibition *in vivo* is the cessation of cell elongation, independent of changes in Cdc42-GTP at cell tips. Moreover, cessation of elongation is accompanied by a decreased rate of cell-volume expansion. Finally, unlike ectopic localization of Cdc42-GTP and actin after Orb6 inhibition, these other important phenotypes are not suppressed by *gef1Δ*.

Orb6 Inhibition Leads to a Strong Decrease in Exocytosis

To further characterize cessation of elongation and decreased cell-volume expansion in Orb6-inhibited cells, we imaged mCherry-tagged beta-glucan synthase subunit Bgs4, a multipass transmembrane protein required for cell wall synthesis at sites of active growth (i.e., cell tips during interphase and the cell midzone during cytokinesis) (Figure S1E) (Cortés et al., 2005, 2015). Within 12–15 min after Orb6 inhibition, Bgs4 was almost completely lost from cell tips in both wild-type (i.e., *gef1+*) and *gef1Δ* backgrounds, although in a small proportion

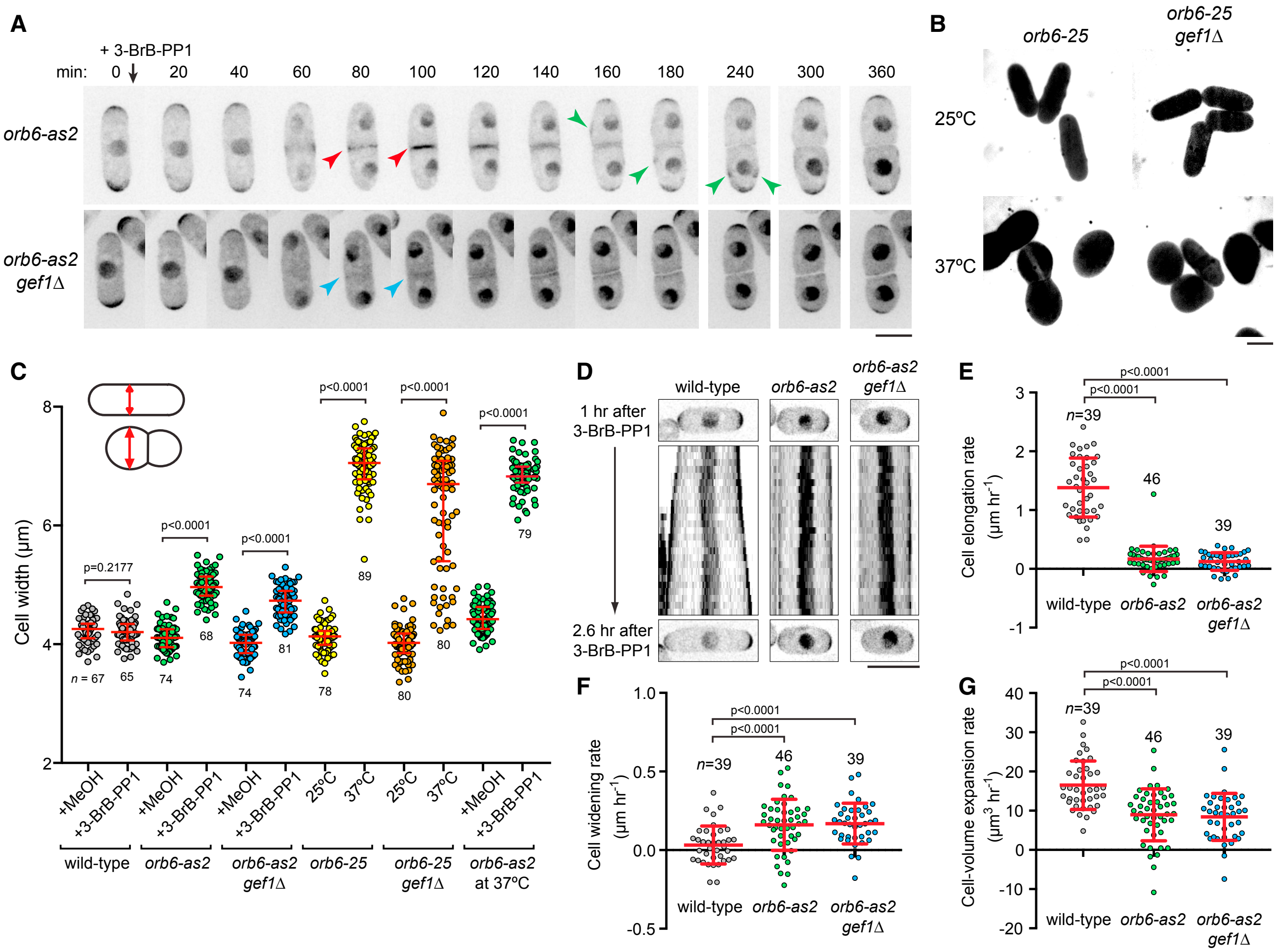

Figure 1. Orb6 Inhibition Leads to Increased Cell Width, Cell-Separation Defects, and Cessation of Polarized Elongation in Both Wild-Type and *gef1Δ* Cells

(A) Video time points of Cdc42-GTP reporter CRIB-3mCitrine after Orb6 inhibition (3-BrB-PP1 treatment of *orb6-as2*) in indicated strains. 3-BrB-PP1 was added after the 0 time point. Both strains show cell-width increase and cell-separation defects. Video S1 shows more cells, with Lifeact-3mCherry. Green arrowheads indicate ectopic CRIB-3mCitrine on cell sides in *orb6-as2* cells, but not *orb6-as2 gef1Δ* cells. Red arrowheads indicate CRIB-3mCitrine at the midzone during early stages of septation in *orb6-as2* cells. Blue arrowheads indicate the absence of CRIB-3mCitrine at comparable stages in *orb6-as2 gef1Δ* cells (Video S1).

(B) Morphology of indicated strains at 25°C and after 5 h at 37°C, shown by fluorescein-dextran exclusion. Images of this type were used for cell-width measurements in (C).

(C) Cell width in indicated strains after 5 h of the indicated treatment and/or condition. Diagrams illustrate how width was measured. Red lines and error bars indicate median and interquartile range.

(D) Kymographs of CRIB-3mCitrine in indicated strains, starting 1 h after 3-BrB-PP1 addition. Video S4 shows the same cells. Orb6-inhibited cells do not elongate, despite Cdc42-GTP enrichment at cell tips.

(E–G) Rates of cell elongation (E), cell widening (F), and cell-volume expansion (G) from experiments as in (D). Red lines and error bars indicate mean and SD. Cell-volume expansion is derived from the other two parameters (see STAR Methods). Videos show that slightly positive values for cell elongation rates in *orb6-as2* and *orb6-as2 gef1Δ* cells are due to cell swelling in all directions and not to polarized elongation (Video S4).

n shows the number of cells scored. Scale bars, 5 μm. p values were determined by two-tailed unpaired Mann-Whitney test. See also Figure S1 and Videos S2 and S3. Three biological replicates were performed for (A) and (D). Measurements for (E)–(G) were made from one of the replicates. Imaging experiments for (B) and (C) were performed once.

of *gef1Δ* cells, some Bgs4 remained either at or just underneath cell tips (Figures 2A, 2B, and 2D; Video S5). Loss of Bgs4 from tips was accompanied by an increase in cytoplasmic Bgs4 puncta, suggesting that Bgs4 was now in endomembrane compartments. Control wild-type (*orb6*$^{+}$) cells treated with 3-BrB-PP1 showed no change in mCherry-Bgs4 localization and continued to elongate (Figures 2A and S1F).

In Orb6-inhibited cells attempting to divide, Bgs4 accumulated at the septation zone for long periods (Figure 2B; Video S5), consistent with observed cell-separation defects. Because

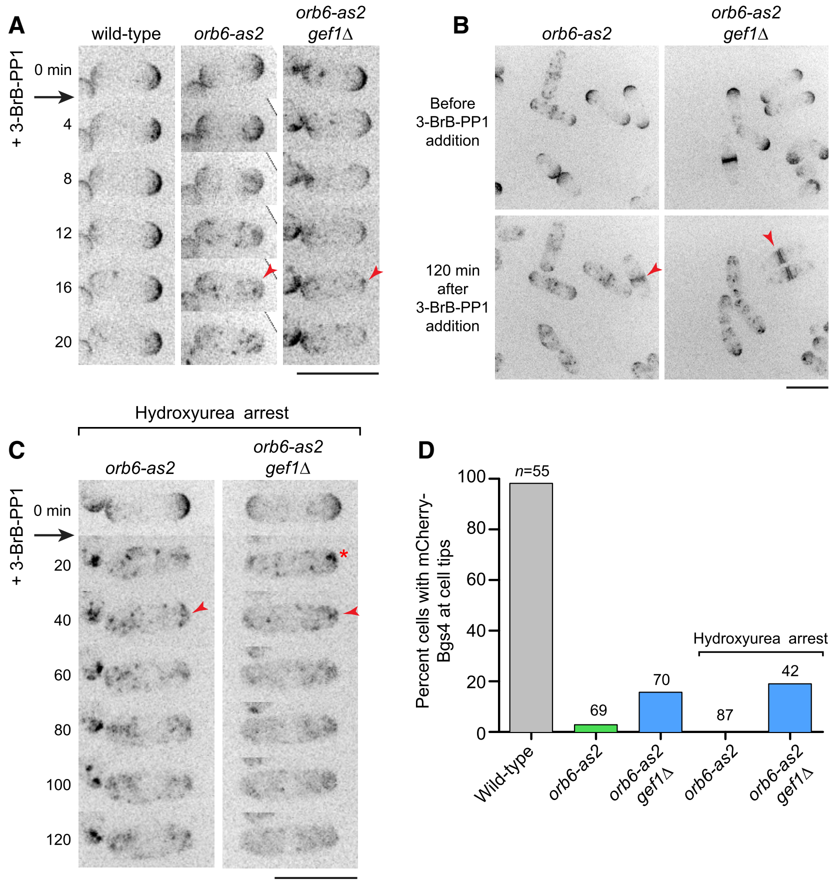

Figure 2. After Orb6 Inhibition, Integral Membrane Protein Bgs4 Is Lost from the Plasma Membrane at Interphase Cell Tips in Both Wild-Type and *gef1Δ* Cells

(A) Video time points of mCherry-Bgs4 after Orb6 inhibition in indicated strains. 3-BrB-PP1 was added after the 0 time point. Arrowheads indicate loss of Bgs4 from cell tips.

(B) Matched fields of cells before and after 3-BrB-PP1 addition to inhibit Orb6. Arrowheads indicate Bgs4 accumulation at the septum. Video S5 shows the same cells.

(C) Video time points of mCherry-Bgs4 after Orb6 inhibition in hydroxyurea-arrested (G1/S) cells. Arrowheads indicate sustained loss of Bgs4 from cell tips, independent of karyokinesis. The asterisk indicates a cluster of endocytosed Bgs4 under the plasma membrane. Video S5 shows more cells, including these.

(D) Quantification of mCherry-Bgs4 cell-tip localization in indicated strains 120 min after 3-BrB-PP1 addition. n shows the number of cells scored. Scale bars, 10 μm. See also Figure S1. Three biological replicates were performed for (A) and (B), and two biological replicates were performed for (C).

nearly all Orb6-inhibited interphase cells eventually progress into mitosis, we wanted to confirm that loss of Bgs4 from cell tips after Orb6 inhibition during interphase was not obligately associated with later recruitment of Bgs4 to the cell midzone during division. We therefore arrested *orb6-as2* or *orb6-as2 gef1Δ* cells in G1/S with hydroxyurea and then inhibited Orb6. In both cases, Bgs4 was quickly lost from cell tips for the duration of imaging (~2 h) (Figures 2C and 2D; Video S5). Collectively, these results suggest that Orb6 kinase activity is essential for maintaining plasma membrane localization of Bgs4, and hence cell elongation, at interphase cell tips.

Because Bgs4 is a multipass transmembrane protein, we would expect its disappearance from cell tips after Orb6 inhibition to be mediated by endocytosis. To confirm this, we blocked endocytosis in *orb6-as2* cells by depolymerizing the actin cytoskeleton with latrunculin A (Ayscough, 2005) and then inhibited Orb6. In these cells, Bgs4 remained at cell tips for at least 2 h after Orb6 inhibition (Figure 3A; Video S6), strongly suggesting that loss of Bgs4 from cell tips after Orb6 inhibition occurs via endocytosis.

These findings suggested that loss of Bgs4 from cell tips after Orb6 inhibition may be due to impaired exocytosis in the presence of continued endocytosis. This would also explain, at least partly, both the cessation of cell elongation during interphase and the cell-separation defects during cell division (due to inability to dissolve the primary septum) (Martín-Cuadrado et al., 2003; Wang et al., 2002). To test whether exocytosis is impaired in Orb6-inhibited cells, we measured secretion of acid phosphatase (ACP) activity (Wang et al., 2002) into the culture medium. In control-treated *orb6-as2* and *orb6-as2 gef1Δ* cells, ACP secretion was similar to that in wild-type cells (Figure 3B). By contrast, in Orb6-inhibited cells (both *gef1*+ and *gef1Δ*), ACP secretion was sharply decreased. (Figure 3B). These results suggest that Orb6 positively regulates exocytosis and that this regulation is independent of Gef1.

In these experiments, we noticed that 3-BrB-PP1-treated wild-type cells showed higher (~4-fold) ACP secretion than control-treated wild-type cells (Figure 3B). In mass spectrometry experiments associated with phosphoproteomics analysis after Orb6 inhibition (described later), we found that 3-BrB-PP1 treatment leads to increased intracellular levels of the major acid phosphatase Pho1 (Figure S2B). Increased Pho1 levels likely account for the increased ACP secretion seen in 3-BrB-PP1-treated wild-type cells as a result of mass action. 3-BrB-PP1 treatment also led to increased intracellular Pho1 levels in *orb6-as2* cells (Figure S2B), even though these cells showed decreased ACP secretion. These results indicate that the decrease in ACP secretion observed in Orb6-inhibited cells is due to a specific decrease in exocytosis and is not simply due to a decrease in ACP levels relative to uninhibited cells.

To further investigate the role of Orb6 in exocytosis, we measured fluorescence recovery after photobleaching (FRAP) of the plasma membrane t-SNARE Psy1 in Orb6-inhibited cells (Figures 3C and 3D). Psy1 is a single-pass transmembrane protein distributed approximately uniformly on the plasma membrane (Bendezú et al., 2015; Maeda et al., 2009). Previous FRAP experiments showed that GFP-Psy1 recovers fluorescence when bleached at cell tips, but not when bleached at cell sides (Bendezú et al., 2015); this implies that Psy1 is not highly mobile

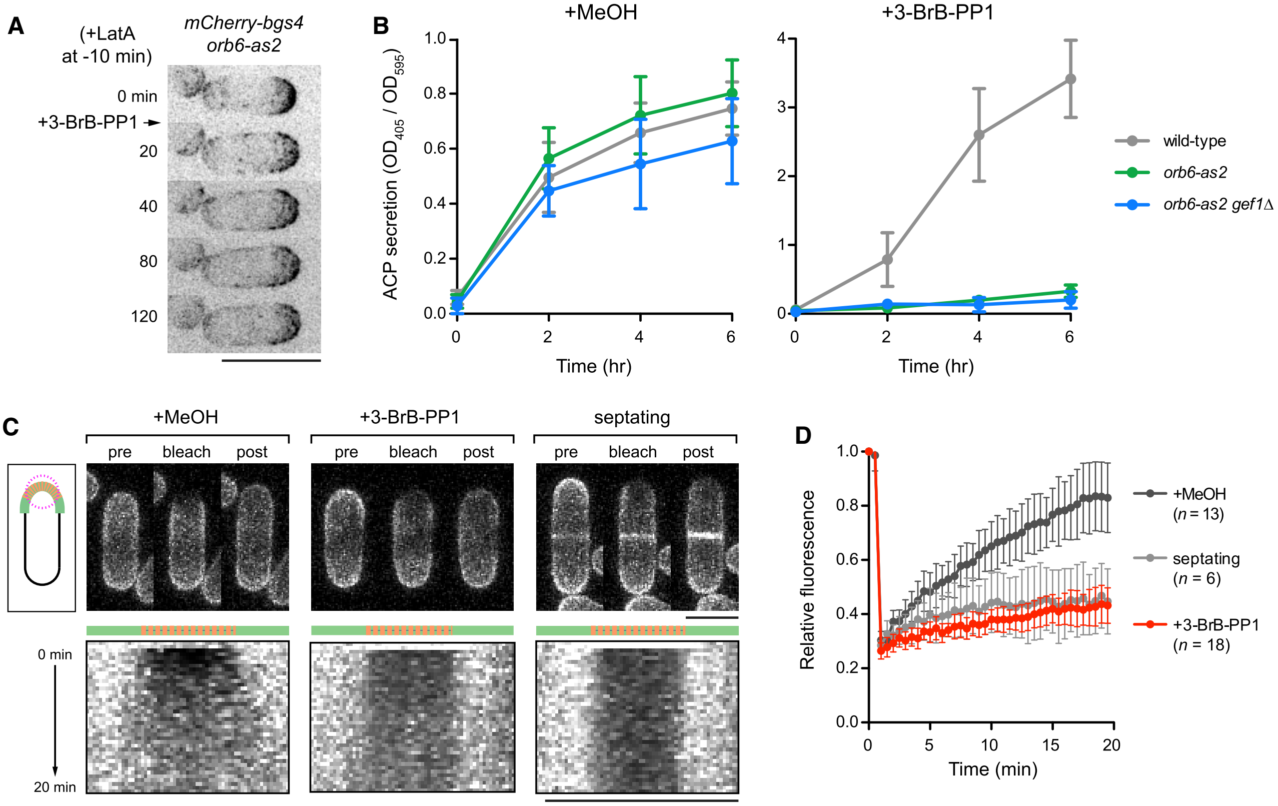

Figure 3. Exocytosis Defects in Wild-Type and *gef1Δ* Cells after Orb6 Inhibition

(A) Video time points of mCherry-Bgs4 after combining actin depolymerization by latrunculin A (LatA) with Orb6 inhibition. Prior disruption of endocytosis by LatA prevents loss of Bgs4 from cell tips after Orb6 inhibition (compare with Figure 2A). Video S6 shows more cells, including this cell, together with *gef1Δ* cells treated in the same way.

(B) Secretion of acid phosphatase (ACP) activity into culture medium in indicated strains after treatment with methanol (left) or 30 μM 3-BrB-PP1 (right). Graphs show mean ACP activity normalized to cell density from replicate experiments. Error bars show SEM.

(C) Fluorescence recovery after photobleaching (FRAP) of plasma membrane t-SNARE GFP-Psy1 at cell tips in *orb6-as2* cells under the indicated conditions. The top-left diagram shows the target bleaching zone (magenta), line scan along the plasma membrane (green), and bleached portion of the plasma membrane (orange). The top images are from representative experiments. The bottom images are corresponding kymographs based on the line scan and bleached portion shown in the diagram.

(D) Quantification of FRAP from experiments as in (C) (see STAR Methods). Symbols indicate mean values. Error bars indicate SD. n indicates the number of cells analyzed.

Scale bars, 10 μm. The imaging experiment for (A) was performed once. For (B), four biological replicates were performed for wild-type and *orb6-as2* cells, and two biological replicates were performed for *orb6-as2 gef1Δ* cells. Two biological replicates of FRAP imaging were performed for (C).

in the plane of the plasma membrane and that Psy1 fluorescence recovery at cell tips is due primarily to turnover via endo- and exocytosis. Consistent with previous work, GFP-Psy1 bleached at control interphase cell tips recovered to approximately 80% of pre-bleaching levels within 20 min after bleaching (Figure 3D), with essentially uniform recovery across the bleached region (Figure 3C). By contrast, GFP-Psy1 bleached at Orb6-inhibited cell tips showed little recovery. As an additional control, we bleached GFP-Psy1 at control cell tips during septation, when membrane trafficking is concentrated at the cell midzone rather than cell tips; in these cells, we also observed little fluorescence recovery. Collectively, these results suggest that decreased fluorescence recovery of GFP-Psy1 at cell tips in Orb6-inhibited cells is due to defects in exocytic membrane trafficking.

We conclude that inhibition of Orb6 kinase activity impairs exocytosis independent of Gef1.

Identification of Orb6 Substrates by Quantitative Global Phosphoproteomics

To date, the only reported substrates of Orb6 are Gef1 and Sts5, both of which were phosphorylated *in vitro* by Orb6 in immunoprecipitates of the Orb6 coactivator Mob2 (Das et al., 2015; Nuñez et al., 2016; Wiley et al., 2003). Sts5 is not known to be associated with exocytosis, and our results indicate that decreased exocytosis after Orb6 inhibition does not involve Gef1. This suggested that other, unknown substrates of Orb6 may regulate exocytosis.

To identify Orb6 substrates, we combined stable isotope labeling with amino acids in culture (SILAC) (Bicho et al., 2010; Macek et al., 2017; Ong and Mann, 2006) with global phosphoproteomics analysis (Figure S2A). We treated light- and heavy-labeled *orb6-as2* cells with either methanol (control) or 3-BrB-PP1 and quantitatively analyzed phosphopeptides by mass spectrometry

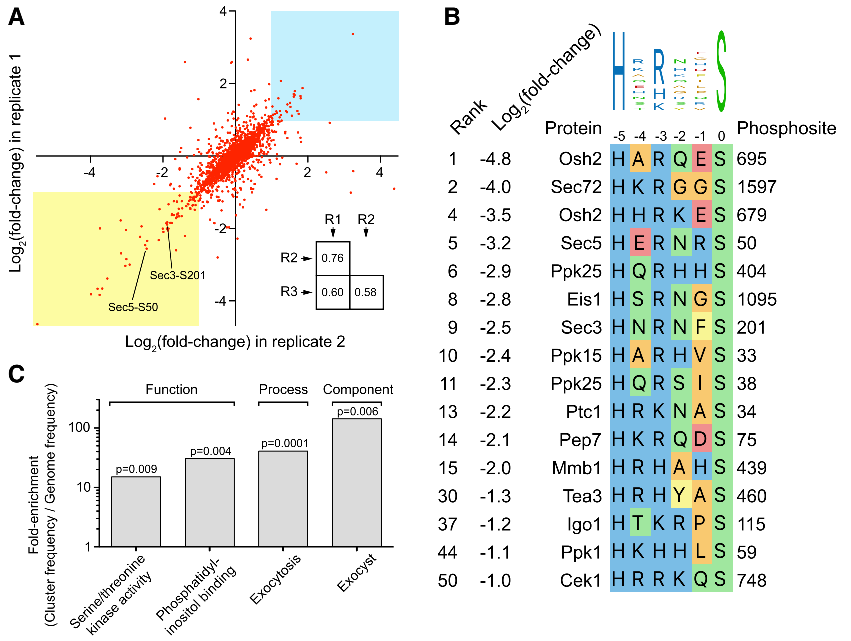

Figure 4. Identification of Orb6 Targets *In Vivo* by Quantitative Global Phosphoproteomics

(A) Correlation of changes in phosphorylation after Orb6 inhibition for 2,415 quantified phosphosites common to two biological replicate experiments (R1 and R2). Axes show log_2(fold change) in phosphorylation of individual phosphosites. The yellow region indicates phosphosites with 2-fold or greater decreased phosphorylation after Orb6 inhibition in both experiments. The blue region indicates relative scarcity of phosphosites with equivalent increase in phosphorylation. Phosphosites Sec3 serine-201 (S201) and Sec5 serine-50 (S50) are indicated. The inset shows Pearson correlation coefficients for three biological replicates (Table S1).

(B) Match of highest-confidence and highest-ranking Orb6-dependent phosphosites to the NDR/LATS consensus (HxR/H/KxxS/T). All indicated phosphosites were quantified in all three biological replicates and showed 2-fold or greater decreased phosphorylation after Orb6 inhibition. Phosphosites were ranked by fold decrease in phosphorylation, independent of whether they matched the NDR/LATS motif (Table S2).

(C) Enrichment of gene ontology terms associated with genes shown in (B), based on molecular function, biological process, and cellular component ontologies. p values were determined by Genetic Gene Ontology (GO) Term Finder.

See also Figures S2 and S3.

to identify phosphosites with decreased phosphorylation after Orb6 inhibition (see STAR Methods). Phosphopeptide abundance was normalized to the abundance of the relevant individual proteins, and to control for possible off-target effects of 3-BrB-PP1, we analyzed phosphopeptides in wild-type cells treated with 3-BrB-PP1.

Overall results from SILAC phosphoproteomics are presented in Table S1. From three biological replicate experiments, we identified 10,866 phosphosites, of which 8,134 phosphosites could be quantified. Among the quantified phosphosites, 326 (4%) showed a 2-fold or greater decrease in phosphorylation after Orb6 inhibition in at least one experiment, 121 (1.5%) showed this decrease in at least two experiments, and 55 (0.7%) showed this decrease in all three experiments (Table S2). Correlation between replicate experiments is shown in Figure 4A.

Because phosphosites with decreased phosphorylation after Orb6 inhibition could be either direct or indirect targets of Orb6, we compared them to the NDR/LATS consensus motif, HxR/H/KxxS/T (Hao et al., 2008; Mazanka et al., 2008; Zhao et al., 2007). Among the 55 phosphosites with significantly decreased phosphorylation in all three experiments, 36 (65%) contained a basic amino acid (histidine, arginine, or lysine) at the −3 position relative to the phosphorylated residue (Figures 4B, S2C, and S2D; Table S2). NDR/LATS kinases are unique among AGC-family kinases (and possibly among all characterized serine-threonine kinases) in their strong preference for histidine at the −5 position (Hao et al., 2008; Pearce et al., 2010). Among the 15 phosphosites with 4-fold or greater decreased phosphorylation in all three experiments, 12 showed a perfect match to the NDR/LATS consensus, including histidine at the −5 position, suggesting that they are direct Orb6 targets (Figure 4B; Table S2).

Gene Ontology (GO) analysis of proteins with the highest-ranking phosphosites matching the NDR/LATS consensus revealed specific enrichment for GO terms related to protein kinase signaling (Cek1, Ppk1, Ppk15, and Ppk25), exocytosis and phosphatidylinositol binding (Pep7, Sec3, Sec5, and Osh2), and the exocyst complex (Sec3 and Sec5) (Figure 4C). Given the importance of the exocyst complex for docking secretory vesicles at the plasma membrane before membrane fusion during exocytosis (Heider and Munson, 2012; Wu and Guo, 2015), we focused further attention on exocyst proteins Sec3 and Sec5, which showed decreased phosphorylation on residues serine-201 and serine-50, respectively.

Orb6 Inhibition Leads to Exocyst Dephosphorylation and Loss from Cell Tips

To confirm Orb6-dependent phosphorylation of Sec3 and Sec5, we combined SILAC with Sec5 immunoprecipitation to analyze exocyst phosphorylation in control versus Orb6-inhibited cells. In Sec5-mCherry immunoprecipitates, levels of all other exocyst proteins (Sec3, Sec6, Sec8, Sec10, Sec15, Exo70, and Exo84) were similar between control and Orb6-inhibited cells, suggesting that exocyst assembly is not grossly affected by Orb6 inhibition (Figures S3A and S3B). Consistent with global phosphoproteomics data, immunoprecipitates from Orb6-inhibited cells showed decreased phosphorylation of Sec3 serine-201 and Sec5 serine-50, as well as decreased phosphorylation of a novel site, Sec8 serine-440 (Figures S3C and S3D; the Sec8 site does not correspond to the NDR/LATS consensus). We also found that unlike Sec5 and Sec8, Sec3 contains numerous additional phosphosites with modestly increased or decreased phosphorylation after Orb6 inhibition (Figures S3C and S3D). No other significant differences in exocyst phosphorylation were observed.

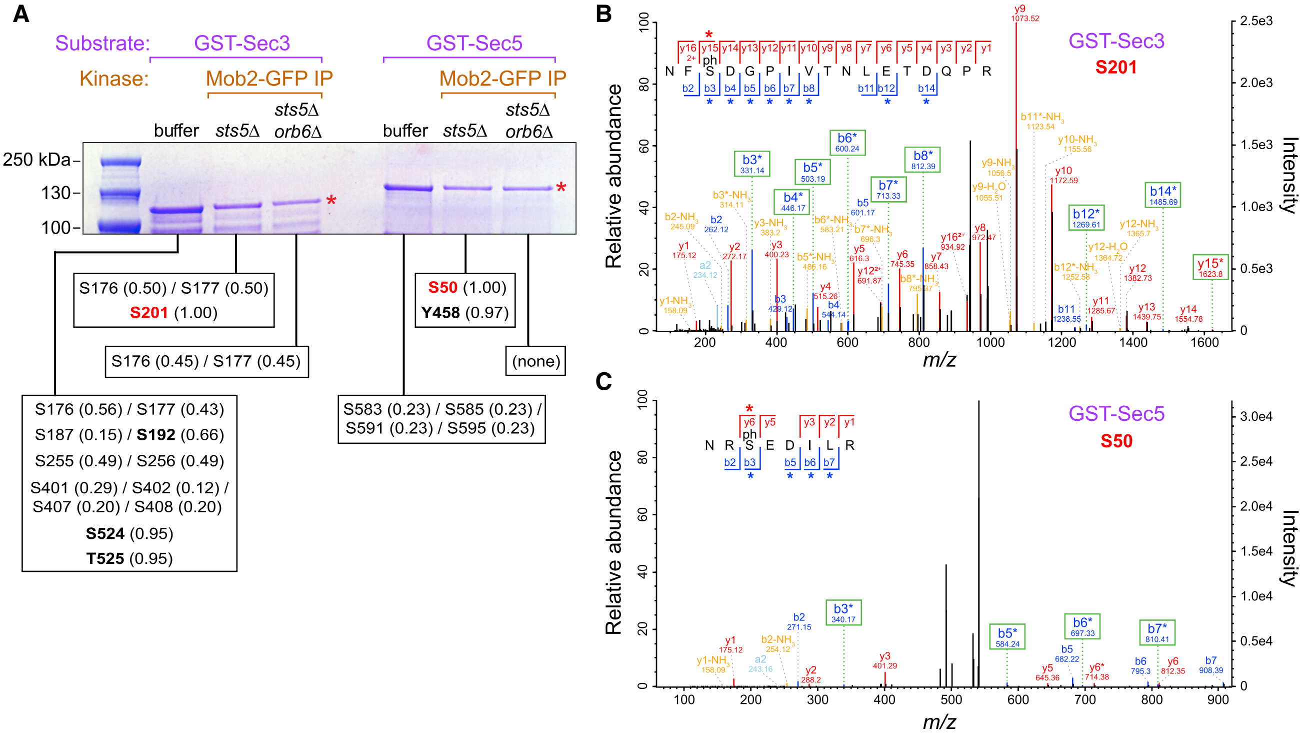

Figure 5. Orb6 Phosphorylates Sec3 and Sec5 *In Vitro*

(A) SDS-PAGE of *in vitro* kinase reactions using recombinant GST-Sec3 or GST-Sec5 (red asterisks) incubated with buffer alone or with Mob2-GFP immunoprecipitates from *sts5Δ* or *sts5Δ orb6Δ* cells. Phosphosites identified by mass spectrometry (MS) in each reaction are shown below, with associated probabilities (in parentheses) for the precise location of the phosphorylated residue. In some cases, the precise location is ambiguous due to incomplete sets of b and y ions. Residues with very high probabilities are shown in bold.

(B and C) Tandem mass spectrometry (MS/MS) spectra of phosphopeptides indicating phosphorylation of Sec3 serine-201 (B) and Sec5 serine-50 (C). b and y ions marked with asterisks indicate neutral loss of 98 Da, an additional diagnostic for the phosphorylated residue. Note different intensity scales in (B) and (C). *In vitro* kinase reactions and phosphomapping were performed once.

To demonstrate Orb6 phosphorylation of Sec3 and Sec5 *in vitro*, we performed kinase assays on bacterially expressed recombinant glutathione S-transferase (GST)-Sec3 and GST-Sec5 using Mob2-GFP immunoprecipitates as a source of active Orb6 (Figure 5A). Because Orb6 is normally essential for viability and viable *orb6Δ* cells can be recovered only in a *sts5Δ* background (Nuñez et al., 2016), we immunoprecipitated Mob2-GFP from *sts5Δ* cells for the kinase reaction and from *sts5Δ orb6Δ* cells as negative control. Consistent with global phosphoproteomics, we identified phosphorylation of Sec3 serine-201 and Sec5 serine-50 in reactions prepared from *sts5Δ* cells, but not from *sts5Δ orb6Δ* cells or buffer-only controls (Figures 5B–5D). These phosphorylations were highly specific, because only one other site (Sec5 tyrosine-458) was phosphorylated *in vitro* in an Orb6-dependent manner (Figure 5B).

We next examined how Orb6 inhibition affects exocyst protein localization *in vivo*. In untreated *orb6-as2* cells, fluorescent-tagged exocyst proteins Sec3, Sec5, Sec8, and Exo70 localized to cell tips in interphase and the septation zone during division, while after Orb6 inhibition, these proteins showed decreased localization at cell tips and ectopic puncta on the peripheral cortex (Figure 6; Video S7). By contrast, 3-BrB-PP1-treated wild-type (*orb6*$^{+}$) cells displayed normal exocyst localization (Figure S4). It is unclear whether decreased exocyst localization at cell tips is a direct consequence of Orb6 inhibition or an indirect consequence (e.g., due to impaired exocytosis). After Orb6 inhibition, exocyst proteins also showed prolonged localization to the septation zone, consistent with cell-separation defects. The cell-separation defect caused by Orb6 inhibition mimics the consequences of inactivating exocyst in fission yeast (Bendezú et al., 2012; Jourdain et al., 2012; Wang et al., 2002).

Altogether, our results indicate that Orb6 phosphorylates exocyst proteins, and Orb6 kinase activity is important for exocyst localization to the cell tip during interphase and for exocyst function in septum dissolution after cytokinesis.

Phosphorylation of Sec3 Serine-201 Is Important for Exocyst Function

To test the role of Sec3 serine-201 and Sec5 serine-50 phosphorylation in exocytosis, we mutated these residues to non-phosphorylatable alanine at their endogenous loci to make the mutants *sec3-S201A* and *sec5-S50A.* Both single mutants, as well as a *sec3-S201A sec5-S50A* double mutant, were viable under normal growth conditions at 25°C and 36°C (Figures 7 and S5A). Because *sec5Δ* cells are inviable (Hayles et al., 2013) and *sec3Δ* cells are viable only when grown at 25°C in the

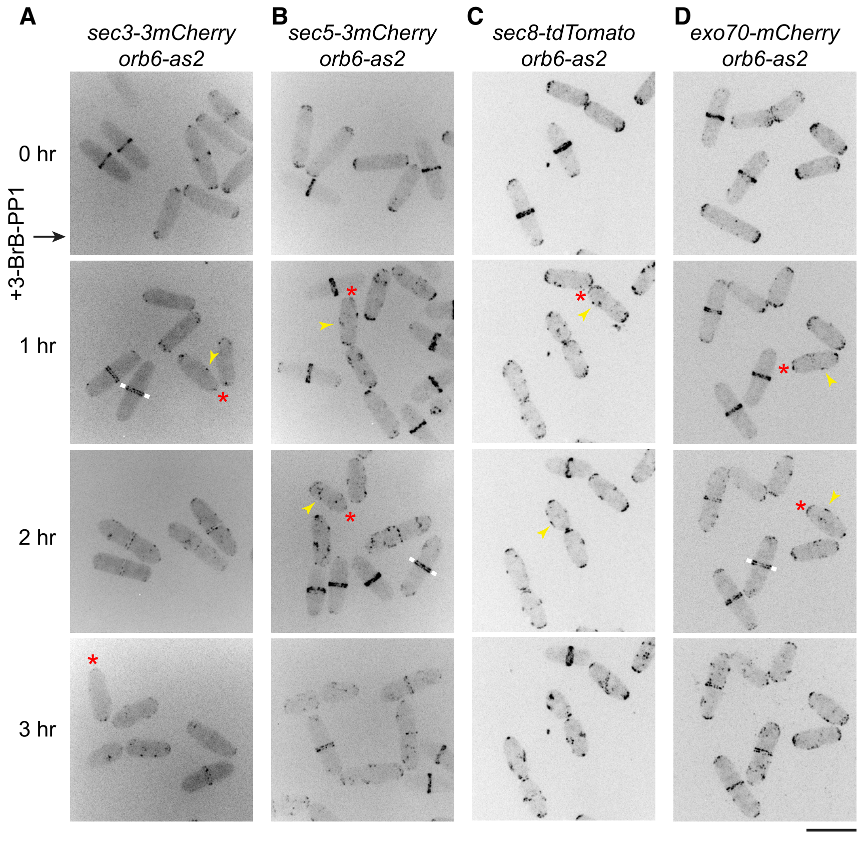

Figure 6. Orb6 Inhibition Leads to Decreased Exocyst at Cell Tips and Ectopic Exocyst at Cell Sides

(A–D) Localization of the indicated fluorescent-tagged exocyst proteins Sec3-3mCherry (A), Sec5-3mCherry (B), Sec8-tdTomato (C), and Exo70-mCherry (D) before and after Orb6 inhibition. 3-BrB-PP1 was added just after the 0 time point. Asterisks indicate decreased or undetectable fluorescence at interphase cell tips after Orb6 inhibition. Arrowheads indicate ectopic exocyst puncta on cell sides. White bars indicate split exocyst localization at the septation zone.

(A and B) Different fields were imaged for each time point to avoid potential photobleaching.

(C and D) Signals were brighter, and the same fields were imaged for all time points.

Video S7 shows changes in Sec3-3mCherry and Sec5-3mCherry localization at a higher time resolution. Scale bar, 10 μm. See also Figure S4. Three biological replicates were performed for (A) and (B). Imaging experiments for (C) and (D) were performed once.

presence of 1 M sorbitol (Bendezú et al., 2012), *sec3-S201A* and *sec5-S50A* are not complete loss-of-function mutations. However, in ACP secretion assays, *sec3-S201A* cells showed impaired secretion relative to wild-type (Figure 7A). The *sec5-S50A* mutation did not affect secretion in either wild-type or *sec3-S201A* backgrounds. This suggests that Sec3 serine-201 phosphorylation, but not Sec5 serine-50 phosphorylation, is important for exocytosis.

Because cell-separation defects are observed in several exocyst deletion mutants (Bendezú et al., 2012; Jourdain et al., 2012; Wang et al., 2002), we assayed the septation index in *sec3-S201A* and *sec5-S50A* mutants. Neither *sec3-S201A* and *sec5-S50A* single mutants nor the double mutant showed an increased septation index relative to wild-type cells (Figures 7C, 7D, and S5A). In addition, both Sec3-S201A-mCherry and Sec5-S50A-mCherry localized to cell tips during interphase and to the septation zone during cell division, like their wild-type counterparts (Figure 7B).

Given these results, we asked whether the function of Sec3 and/or Sec5 phosphorylation overlaps with other exocyst components, in particular Exo70. In fission yeast, *sec3* and *exo70* are redundant for viability: *sec3Δ* and *exo70Δ* mutations are each conditional lethal, but *exo70Δ sec3Δ* double mutants are dead under all conditions (Bendezú et al., 2012). We combined *sec3-S201A* and *sec5-S50A* single and double mutants with *exo70Δ* and assayed ACP secretion and the septation index. All mutant combinations were viable at 25°C and 36°C (Figures 7 and S5A). In ACP secretion assays, all mutants combined with *exo70Δ* behaved similarly to mutants in a wild-type (*exo70+*) background (Figure 7A). However, the septation index was significantly increased in *exo70Δ sec3-S201A* double mutants compared to either single mutant at both 25°C and 36°C; more than 75% of cells contained at least one septum, and 25% of cells contained multiple septa (Figures 7C, 7D, and S5A). This was not altered by further deletion of *gef1* (Figures S5B and S5C). The septation index in *exo70Δ* and *exo70Δ sec3-S201A* mutants was not greatly altered by the *sec5-S50A* mutation, although there were somewhat fewer multi-septate *exo70Δ sec3-S201A sec5-S50A* cells at 25°C compared to 36°C (Figures 7C, 7D, and S5A); the reasons for this are not yet clear. Collectively, these observations suggest that phosphorylation of Sec3 serine-201 by Orb6 works with Exo70 to promote exocyst function in cell separation.

We hypothesized that cell-separation defects in *exo70Δ sec3-S201A* cells might be due to Sec3-S201A failing to localize correctly in the absence of Exo70. However, because spores of the double-mutant *exo70Δ sec3-S201A-3mCherry* failed to germinate (Figure S5D), we were unable to test this directly. We therefore examined Sec5 and Sec8 localization in *exo70Δ sec3-S201A* cells. Both proteins localized normally to cell tips and the septation zone, indicating that exocyst localization per se was not adversely affected (Figures S5E and S5F).

In parallel with these experiments, we constructed *sec3-S201D* and *sec5-S50D* single and double mutants, as well as *sec3-S201E* and *sec5-S50E* single and double mutants, to test whether such phosphomimetic mutations could abrogate the effects of Orb6 inhibition on exocytosis. Imaging of mCherry-Bgs4 in Orb6-inhibited cells containing phosphomimetic *sec3* and/or *sec5* mutations showed that this was not the case (Video S8).

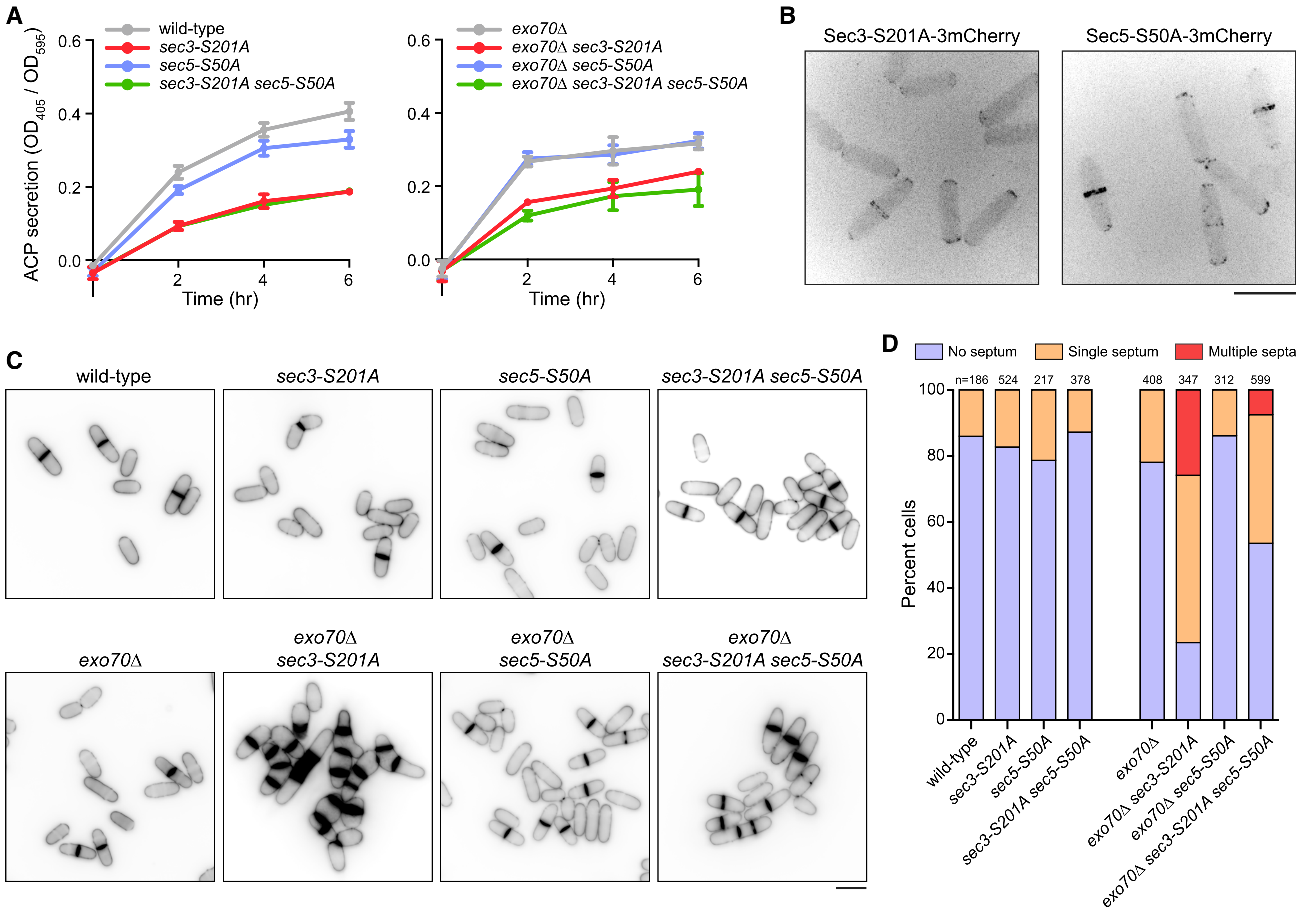

Figure 7. Sec3 Serine-201 Phosphorylation Contributes to Exocyst Function

(A) Secretion of ACP activity into culture medium, normalized to cell density, for indicated strains. Symbols indicate mean values. Error bars indicate SEM. Error bars smaller than datapoint symbols are not shown.

(B) Localization of 3mCherry-tagged Sec3-S201A and Sec5-S50A to cell tips and the septation zone.

(C) Calcofluor staining of indicated strains, showing cell-separation defects in *exo70Δ sec3-S201A* and *exo70Δ sec3-S201A sec5-S50A* cells.

(D) Septation index in cells of indicated strains as in (C).

Scale bars, 10 μm. See also Figure S5 and Video S8. Three biological replicates were performed for ACP assays in (A). Imaging experiments for (B) and (C) were performed once.

After Orb6 inhibition in the phosphomimetic mutants, Bgs4 was quickly lost from cell tips, and cell elongation ceased, as in wild-type cells. It is possible that phosphomimetic *sec3* and *sec5* mutations do not fully reproduce the properties of the naturally phosphorylated residues (Dephoure et al., 2013). Alternatively, the collective effects of Orb6 inhibition on other Orb6 targets (see Discussion) may be sufficient to inhibit exocytosis even when Sec3 and Sec5 remain in a phosphorylated or phosphomimetic state.

DISCUSSION

Here we have shown that the conserved NDR/LATS kinase Orb6 has a major role in regulating fission yeast exocytosis. Round and/or wide cell-polarity defects in *orb6* mutants were previously attributed to failure to spatially restrict activity of Cdc42 GEF Gef1 (Das et al., 2009). However, we find that these and several other *orb6* phenotypes persist in *gef1Δ* cells. Upon inhibition of Orb6 kinase activity *in vivo*, impaired exocytosis is a key Gef1-independent phenotype, and from an unbiased quantitative global phosphoproteomics analysis, we identified several novel Orb6 targets related to membrane trafficking, including proteins implicated in signaling, lipid binding, and exocytosis. Through more detailed analysis of Orb6 phosphorylation of the exocyst complex, we have shown that Sec3 serine-201 phosphorylation is important for exocyst function.

Role of Gef1 in Mediating Orb6 Function

Orb6 inactivation, using either temperature-sensitive *orb6-25* or analog-sensitive *orb6-as2*, results in several phenotypes, including partial ectopic localization of Cdc42-GTP to cell sides (without complete loss from cell tips), actin disorganization, and increased cell width and/or rounding. Cdc42 is fundamental to multiple aspects of cell polarity, including actin organization

and exocyst function (Martin and Arkowitz, 2014); thus, it has been proposed that ectopic Cdc42-GTP localization is a critical mediator of *orb6* phenotypes (Das et al., 2009). While we confirmed that ectopic Cdc42-GTP localization after Orb6 inhibition requires Gef1 (Das et al., 2009), we find that ectopic Cdc42-GTP is not the cause of increased cell width in *orb6* cells, because cell-width increases after Orb6 inactivation in wild-type (*gef1*+) cells are similar to those seen in *gef1Δ* cells, which do not have ectopic Cdc42-GTP. Our results disagree with earlier work reporting smaller width increases in *gef1Δ* cells compared to *gef1*+ cells after Orb6 inactivation (Das et al., 2009). We do not have an explanation for these different findings, which led us to investigate alternative mechanisms by which Orb6 could regulate polarized growth (described later).

Orb6 and Exocytosis

In addition to the phenotypes described earlier, Orb6-inhibited cells (both *gef1*+ and *gef1Δ*) are strongly impaired in exocytosis and cease interphase cell elongation, despite enriched Cdc42-GTP at cell tips. The exocytosis and elongation phenotypes are likely related, as evidenced by disappearance of Bgs4 from cell tips after Orb6 inhibition. Bgs4 disappearance from tips is due to continued endocytosis in the absence of exocytosis, and because Bgs4 is required for cell wall synthesis at cell tips, this can account, at least partly, for cessation of cell elongation.

In Orb6-inhibited cells, although exocytic trafficking of Bgs4 to cell tips is abolished during interphase, Bgs4 accumulates at the cell midzone during cytokinesis on either side of the primary septum. This indicates that during cytokinesis, exocytosis proceeds independently of Orb6 to facilitate processes such as primary septum formation. Later, as cells progress to the next interphase, Orb6-dependent exocytosis is required for secretion of hydrolases to dissolve the primary septum and separate the daughter cells. Our finding that Orb6 regulates exocyst (described later) is consistent with exocyst mutants displaying cell-separation defects, rather than cytokinesis defects (Bendezú et al., 2012; Jourdain et al., 2012; Wang et al., 2002). This, in turn, implies that targeting of secretion toward the midzone during cytokinesis (e.g., for primary septum formation) involves mechanisms other than exocyst (Wang et al., 2002). Based on previous work showing redundancy of exocyst function and actin cable-based vesicle delivery in promoting efficient exocytosis (Bendezú and Martin, 2011; Nakano et al., 2011; Snaith et al., 2011), we speculate that during cytokinesis, the abundance of actin cables contributing to the cytokinetic actomyosin ring (Mishra et al., 2014; Willet et al., 2015) can bypass a need for exocyst.

The cell-separation defect of Orb6-inhibited cells is suppressed by deletion of *sts5*, which encodes an RNA-binding protein (Nuñez et al., 2016). It has been proposed that Orb6 negatively regulates the recruitment of Sts5 into ribonucleoprotein granules and processing bodies (P-bodies), thereby controlling the translational repression of Sts5-associated mRNAs encoding proteins involved in polarized growth and cell separation (Nuñez et al., 2016). Because our data suggest that cell-separation defects in Orb6-inhibited cells are due to defects in exocytosis, it is plausible that *sts5Δ* causes an upregulation in exocytosis post-cytokinesis, promoting dissolution of the primary septum and thus cell separation. Potential links between Sts5 and exocytosis represent an interesting area for future exploration.

Phosphoregulation of Exocyst

We demonstrated phosphorylation of exocyst Sec3 serine-201 and Sec5 serine-50 by Orb6 in multiple assays. While *sec5-S50A* mutants have no detectable phenotype, *sec3-S201A* mutants show clear defects in exocytosis, even though *sec3-S201A* phenotypes are not as severe as *sec3Δ* phenotypes (Bendezú et al., 2012). In addition, double-mutant analysis indicates that the function of Sec3 serine-201 phosphorylation overlaps with that of Exo70 during cell separation.

At a mechanistic level, it is not yet clear what this function is. In both budding and fission yeast, Sec3 and Exo70 are redundantly required for exocyst localization to the plasma membrane, through interactions with phosphatidylinositol 4,5-bisphosphate (PIP2) on the plasma membrane (Bendezú et al., 2012; He et al., 2007; Liu et al., 2007; Zhang et al., 2008). While this raised the possibility that Sec3 serine-201 phosphorylation might be important for exocyst localization, both Sec5 and Sec8 can localize to cell tips and the septation zone in *sec3-S201A exo70Δ* cells, and Sec3-S201A-3mCherry localizes to cell tips and to the septation zone. Thus, Sec3 serine-201 phosphorylation is not required for exocyst localization.

We speculate that Sec3 serine-201 phosphorylation may modulate exocyst conformational dynamics (Heider and Munson, 2012) and/or interaction with small GTPases (Wu et al., 2008). Exocyst is regulated by Rho- and Rab-family GTPases, as well as by protein kinases, and this regulation can alter dynamic aspects of exocyst assembly and/or local activation (Wu et al., 2008; Wu and Guo, 2015). A cryo-electron microscopy (cryo-EM) structure of budding yeast exocyst was unable to model the N-terminal region (~600 amino acids) of Sec3, suggesting that this region may be more disordered or flexible than the bulk of the exocyst complex (Mei et al., 2018; Lepore et al., 2018). Because budding yeast and fission yeast Sec3 have only limited sequence similarity (Bendezú et al., 2012) and Sec3 serine-201 is not obviously conserved in budding yeast, it is difficult to extrapolate directly from one system to the other. However, in this context, the budding yeast Sec3 N terminus contains four NDR/LATS consensus sites, at least two of which are phosphorylated *in vivo* (https://www.yeastgenome.org/locus/S000000810/protein). Like budding yeast Sec3, fission yeast Sec3 contains an N-terminal cryptic pleckstrin homology (PH) domain (residues 1–133) involved in PIP2 binding (Bendezú et al., 2012), but serine-201 lies outside this domain. Additional work will be needed to determine how fission yeast Sec3 serine-201 phosphorylation regulates exocyst.

Additional Orb6 Targets

Both mass spectrometry (MS) analysis and cell phenotypes suggest that Orb6 targets involved in exocytosis are not restricted to exocyst proteins. Orb6 inhibition *in vivo* leads to cessation of cell elongation and decreased exocyst at cell tips, whereas *sec3-S201A exo70Δ* cells, despite impaired ACP

secretion and cell-separation defects, can grow in a polarized fashion and maintain exocyst at cell tips (Figures 7 and S5). We propose that the strong phenotypes seen after Orb6 inhibition are due to the total combined changes in phosphorylation of multiple Orb6 targets in several related pathways. In addition to Sec3 and Sec5, Orb6 targets identified in this work include protein kinases and proteins involved in membrane trafficking. Nearly all of the highest-ranking phosphosites (i.e., those with highest reproducibility and greatest fold decrease after Orb6 inhibition) match the NDR/LATS consensus (Figures 4 and S2; Table S2), suggesting that they are direct substrates of Orb6.

In addition to exocyst, we identified Sec72, Pep7, Ppk25, and Osh2 as Orb6 targets. Sec72 is a homolog of budding yeast Sec7, a GEF for Arf1 ADP ribosylation factor that is important for coat formation on nascent vesicles emanating from the Golgi and the trans-Golgi network (Wright et al., 2014). Budding yeast Pep7 (also called Vac1/Vps19) integrates Rab GTPase and phosphatidylinositol 3-kinase signaling and promotes Golgi-to-endosome transport (Peterson et al., 1999). The kinase Ppk25 does not have extensive regulatory domains, but its kinase domain is most similar to that of the budding yeast paralogs Kin1 and Kin2, which function in late stages of exocytosis through phosphorylation of t-SNARE Sec9 (Elbert et al., 2005). Budding yeast Osh2, a member of a conserved family of oxysterol-binding protein-related proteins (Raychaudhuri and Prinz, 2010), facilitates endocytosis at plasma membrane regions that contact cortical endoplasmic reticulum (Encinar Del Dedo et al., 2017). Collectively, the functions of these proteins in budding yeast suggest that in fission yeast, Orb6 coordinates multiple processes related to membrane trafficking.

Although we identified several phosphosites in the previously characterized Orb6 targets Gef1 and Sts5, none of these sites showed significant decreased phosphorylation after Orb6 inhibition (i.e., 2-fold or greater) (Table S1). In addition, although Gef1 serine-112 was previously described as an Orb6-dependent phosphosite *in vitro* (Das et al., 2015), we did not identify it as a phosphosite *in vivo*. However, absence from our datasets does not necessarily imply that Gef1 and Sts5 are not bona fide Orb6 substrates, because global phosphoproteomics analysis is unlikely to be exhaustive.

The notion that Orb6 may have multiple roles in regulating membrane trafficking and cell polarity is consistent with analysis of NDR1/2 kinase function in mammalian cells. NDR1 negatively regulates dendrite length and branching via phosphorylation of targets including AAK1 and Rabin8 (GEF for GTPase Rab8), which regulate vesicle-mediated trafficking (Ultanir et al., 2012). Independently, NDR2 was shown to phosphorylate Rabin8 to promote Rabin8-Sec15 interaction, activating Rab8 during ciliogenesis (Chiba et al., 2013). NDR2 also regulates β1-integrin exocytosis, thus controlling integrin activation for neurite growth and guidance (Rehberg et al., 2014). In addition, in budding yeast, Cbk1 has been proposed to regulate more than one step in the secretory pathway (Kurischko et al., 2008), although some aspects of this may be controversial (Jansen et al., 2009). Collectively, our results suggest that regulation of exocytosis and/or secretion by NDR/LATS kinases may be a generally conserved design principle, although specific targets of NDR/LATS kinases may differ in different systems.

Physiological Function of Switching off Orb6 Activity

Orb6 is the most downstream kinase of the fission yeast morphogenesis network (MOR), a signaling pathway similar in design to the fission yeast septation initiation network (SIN) (Simanis, 2015), budding yeast mitotic exit network (MEN) and regulation of Ace2 and morphogenesis (RAM) network (Weiss, 2012), and metazoan Hippo pathway (Hansen et al., 2015). In addition to Orb6, MOR proteins include Sog2, Mob2, Mor2, and Pmo25, as well as the germinal-center kinase Nak1; all of these are conserved, and all are important for regulation of cell polarity and cell separation (Kanai et al., 2005). MOR signaling decreases during mitosis, when cells switch polarity from tip growth to the midzone for cell division, and it increases again after completion of cytokinesis, coincident with cell separation (Kanai et al., 2005; Ray et al., 2010). Attenuation of MOR signaling in mitosis depends on SIN, and there is compelling evidence that mutual antagonism between MOR and SIN is critical for coordinating changes in cell polarity and cytoskeletal reorganization through the cell cycle (Ray et al., 2010). At the same time, the targets of MOR that execute these changes have generally remained obscure. Based on the results presented here, we propose a simple model in which Orb6 regulation of exocytosis is a key output of MOR signaling. According to this view, during interphase, Orb6 promotes polarized exocytosis at cell tips, while during mitosis, decreased Orb6 activity shuts down exocytosis at tips, allowing Orb6-independent redistribution of secretion toward the cell midzone. After cytokinesis (effectively, the next interphase), the return of Orb6 activity specifically promotes exocytosis of hydrolases into the septation zone, dissolving the primary septum for daughter-cell separation and allowing resumption of growth at cell ends. While further functional characterization of Orb6 targets awaits elucidation, we speculate that by coordinating multiple kinase signaling and vesicle-mediated trafficking pathways, Orb6 may function as an interphase-specific master regulator of membrane trafficking and exocytosis, thus helping to drive transitions in cellular organization between interphase and mitosis.

STAR★METHODS

Detailed methods are provided in the online version of this paper and include the following:

- KEY RESOURCES TABLE
- CONTACT FOR REAGENT AND RESOURCE SHARING
- EXPERIMENTAL MODEL AND SUBJECT DETAILS
  - Fission Yeast Strains and Construction
- METHODS DETAILS
  - Yeast culture
  - Plasmid construction
  - Microscopy sample preparation and imaging
  - Microscopy image analysis
  - ACP assay
  - Sample preparation for global phosphoproteomics
  - Peptide fractionation
  - Phosphopeptide enrichment
  - In-gel digestion for MS
  - LC-MS/MS

- ○ Global phosphoproteomics data analysis and presentation
- ○ Immunoprecipitation of Sec5-3mCherry
- ○ Purification of GST-Sec3 and GST-Sec5
- ○ *In vitro* kinase assays
- ● QUANTIFICATION AND STATISTICAL ANALYSIS
  - ○ Statistical tests
- ● DATA AND SOFTWARE AVAILABILITY

SUPPLEMENTAL INFORMATION

Supplemental Information includes five figures, three tables, and eight videos and can be found with this article online at https://doi.org/10.1016/j.celrep.2019.01.027.

ACKNOWLEDGMENTS

We thank M. Balasubramanian, D. McCollum, T. Nakamura, P. Nurse, F. Verde, P. Perez, J.C. Ribas, and H. Snaith for strains; I. Hagan and A. Patel for advice and protocols on phosphoproteomics; H. Thakur and S.L. Leong for advice on protein purification; and members of our laboratories for discussion. This work was supported by the BBRSC (BB/K021699/1 to K.E.S. and A.B.G.) and by the Wellcome Trust (094517 and 210659 to K.E.S., 108504 and 091020 to J.R., and a multi-user equipment grant 101527 for the Edinburgh Protein Production Facility). The Wellcome Centre for Cell Biology is supported by core funding from the Wellcome Trust (203149).

AUTHOR CONTRIBUTIONS

Y.D.T., M.L., A.B.G., and K.E.S. conceived and designed the experiments. Y.D.T., M.L., and C.S. performed and analyzed the experiments. M.L., C.S., and J.R. contributed analysis tools. Y.D.T., C.S., and K.S. wrote the manuscript. Y.D.T., M.L., C.S., J.R., A.B.G., and K.E.S. edited/reviewed the manuscript.

DECLARATION OF INTERESTS

The authors declare no competing interests.

Received: June 20, 2018
Revised: November 3, 2018
Accepted: January 8, 2019
Published: February 5, 2019

REFERENCES

Ayscough, K.R. (2005). Coupling actin dynamics to the endocytic process in *Saccharomyces cerevisiae*. Protoplasma *226*, 81–88.

Bähler, J., Wu, J.Q., Longtine, M.S., Shah, N.G., McKenzie, A., 3rd, Steever, A.B., Wach, A., Philippsen, P., and Pringle, J.R. (1998). Heterologous modules for efficient and versatile PCR-based gene targeting in *Schizosaccharomyces pombe*. Yeast *14*, 943–951.

Bendezú, F.O., and Martin, S.G. (2011). Actin cables and the exocyst form two independent morphogenesis pathways in the fission yeast. Mol. Biol. Cell *22*, 44–53.

Bendezú, F.O., Vincenzetti, V., and Martin, S.G. (2012). Fission yeast Sec3 and Exo70 are transported on actin cables and localize the exocyst complex to cell poles. PLoS ONE *7*, e40248.

Bendezú, F.O., Vincenzetti, V., Vavylonis, D., Wyss, R., Vogel, H., and Martin, S.G. (2015). Spontaneous Cdc42 polarization independent of GDI-mediated extraction and actin-based trafficking. PLoS Biol. *13*, e1002097.

Bicho, C.C., de Lima Alves, F., Chen, Z.A., Rappsilber, J., and Sawin, K.E. (2010). A genetic engineering solution to the "arginine conversion problem" in stable isotope labeling by amino acids in cell culture (SILAC). Mol. Cell. Proteomics *9*, 1567–1577.

Bidlingmaier, S., Weiss, E.L., Seidel, C., Drubin, D.G., and Snyder, M. (2001). The Cbk1p pathway is important for polarized cell growth and cell separation in *Saccharomyces cerevisiae*. Mol. Cell. Biol. *21*, 2449–2462.

Bishop, A.C., Ubersax, J.A., Petsch, D.T., Matheos, D.P., Gray, N.S., Blethrow, J., Shimizu, E., Tsien, J.Z., Schultz, P.G., Rose, M.D., et al. (2000). A chemical switch for inhibitor-sensitive alleles of any protein kinase. Nature *407*, 395–401.

Campanale, J.P., Sun, T.Y., and Montell, D.J. (2017). Development and dynamics of cell polarity at a glance. J. Cell Sci. *130*, 1201–1207.

Chiba, S., Amagai, Y., Homma, Y., Fukuda, M., and Mizuno, K. (2013). NDR2-mediated Rabin8 phosphorylation is crucial for ciliogenesis by switching binding specificity from phosphatidylserine to Sec15. EMBO J. *32*, 874–885.

Chiyoda, T., Sugiyama, N., Shimizu, T., Naoe, H., Kobayashi, Y., Ishizawa, J., Arima, Y., Tsuda, H., Ito, M., Kaibuchi, K., et al. (2012). LATS1/WARTS phosphorylates MYPT1 to counteract PLK1 and regulate mammalian mitotic progression. J. Cell Biol. *197*, 625–641.

Cornils, H., Kohler, R.S., Hergovich, A., and Hemmings, B.A. (2011). Human NDR kinases control G(1)/S cell cycle transition by directly regulating p21 stability. Mol. Cell. Biol. *31*, 1382–1395.

Cortés, J.C., Carnero, E., Ishiguro, J., Sánchez, Y., Durán, A., and Ribas, J.C. (2005). The novel fission yeast (1,3)beta-D-glucan synthase catalytic subunit Bgs4p is essential during both cytokinesis and polarized growth. J. Cell Sci. *118*, 157–174.

Cortés, J.C., Pujol, N., Sato, M., Pinar, M., Ramos, M., Moreno, B., Osumi, M., Ribas, J.C., and Pérez, P. (2015). Cooperation between paxillin-like protein Pxl1 and glucan synthase Bgs1 is essential for actomyosin ring stability and septum formation in fission yeast. PLoS Genet. *11*, e1005358.

Cox, J., and Mann, M. (2008). MaxQuant enables high peptide identification rates, individualized p.p.b.-range mass accuracies and proteome-wide protein quantification. Nat. Biotechnol *26*, 1367–1372.

Cox, J., Neuhauser, N., Michalski, A., Scheltema, R.A., Olsen, J.V., and Mann, M. (2011). Andromeda: a peptide search engine integrated into the MaxQuant environment. J. Proteome Res. *10*, 1794–1805.

Das, M., Wiley, D.J., Chen, X., Shah, K., and Verde, F. (2009). The conserved NDR kinase Orb6 controls polarized cell growth by spatial regulation of the small GTPase Cdc42. Curr. Biol. *19*, 1314–1319.

Das, M., Nuñez, I., Rodriguez, M., Wiley, D.J., Rodriguez, J., Sarkeshik, A., Yates, J.R., 3rd, Buchwald, P., and Verde, F. (2015). Phosphorylation-dependent inhibition of Cdc42 GEF Gef1 by 14-3-3 protein Rad24 spatially regulates Cdc42 GTPase activity and oscillatory dynamics during cell morphogenesis. Mol. Biol. Cell *26*, 3520–3534.

Dephoure, N., Gould, K.L., Gygi, S.P., and Kellogg, D.R. (2013). Mapping and analysis of phosphorylation sites: a quick guide for cell biologists. Mol. Biol. Cell *24*, 535–542.

Ekwall, K., and Thon, G. (2017). Spore analysis and tetrad dissection of *Schizosaccharomyces pombe*. Cold Spring Harb. Protoc. *2017*, prot091710. PubMed.

Elbert, M., Rossi, G., and Brennwald, P. (2005). The yeast par-1 homologs kin1 and kin2 show genetic and physical interactions with components of the exocytic machinery. Mol. Biol. Cell *16*, 532–549.

Encinar Del Dedo, J., Idrissi, F.Z., Fernandez-Golbano, I.M., Garcia, P., Rebollo, E., Krzyzanowski, M.K., Grötsch, H., and Geli, M.I. (2017). ORP-mediated ER contact with endocytic sites facilitates actin polymerization. Dev. Cell *43*, 588–602.

Forsburg, S.L., and Rhind, N. (2006). Basic methods for fission yeast. Yeast *23*, 173–183.

Furth, N., and Aylon, Y. (2017). The LATS1 and LATS2 tumor suppressors: beyond the Hippo pathway. Cell Death Differ. *24*, 1488–1501.

Gruhler, A., Olsen, J.V., Mohammed, S., Mortensen, P., Faergeman, N.J., Mann, M., and Jensen, O.N. (2005). Quantitative phosphoproteomics applied to the yeast pheromone signaling pathway. Mol. Cell. Proteomics *4*, 310–327.

Hagan, I.M. (2016). Chromatin and cell wall staining of *Schizosaccharomyces pombe*. Cold Spring Harb. Protoc. *2016*, prot091025. PubMed.

Hansen, C.G., Moroishi, T., and Guan, K.L. (2015). YAP and TAZ: a nexus for Hippo signaling and beyond. Trends Cell Biol. *25*, 499–513.

Hao, Y., Chun, A., Cheung, K., Rashidi, B., and Yang, X. (2008). Tumor suppressor LATS1 is a negative regulator of oncogene YAP. J. Biol. Chem. *283*, 5496–5509.

Hayles, J., Wood, V., Jeffery, L., Hoe, K.L., Kim, D.U., Park, H.O., Salas-Pino, S., Heichinger, C., and Nurse, P. (2013). A genome-wide resource of cell cycle and cell shape genes of fission yeast. Open Biol. *3*, 130053.

He, B., Xi, F., Zhang, X., Zhang, J., and Guo, W. (2007). Exo70 interacts with phospholipids and mediates the targeting of the exocyst to the plasma membrane. EMBO J. *26*, 4053–4065.

Heider, M.R., and Munson, M. (2012). Exorcising the exocyst complex. Traffic *13*, 898–907.

Hergovich, A. (2013). Regulation and functions of mammalian LATS/NDR kinases: looking beyond canonical Hippo signalling. Cell Biosci. *3*, 32.

Hergovich, A. (2016). The roles of NDR protein kinases in Hippo signalling. Genes (Basel) *7*, E21.

Hergovich, A., Stegert, M.R., Schmitz, D., and Hemmings, B.A. (2006). NDR kinases regulate essential cell processes from yeast to humans. Nat. Rev. Mol. Cell Biol. *7*, 253–264.

Ishihama, Y., Rappsilber, J., Andersen, J.S., and Mann, M. (2002). Microcolumns with self-assembled particle frits for proteomics. J. Chromatogr. A. *979*, 233–239.

Jansen, J.M., Wanless, A.G., Seidel, C.W., and Weiss, E.L. (2009). Cbk1 regulation of the RNA-binding protein Ssd1 integrates cell fate with translational control. Curr. Biol. *19*, 2114–2120.

Jourdain, I., Dooley, H.C., and Toda, T. (2012). Fission yeast sec3 bridges the exocyst complex to the actin cytoskeleton. Traffic *13*, 1481–1495.

Kanai, M., Kume, K., Miyahara, K., Sakai, K., Nakamura, K., Leonhard, K., Wiley, D.J., Verde, F., Toda, T., and Hirata, D. (2005). Fission yeast MO25 protein is localized at SPB and septum and is essential for cell morphogenesis. EMBO J. *24*, 3012–3025.

Kee, Y., Yoo, J.S., Hazuka, C.D., Peterson, K.E., Hsu, S.C., and Scheller, R.H. (1997). Subunit structure of the mammalian exocyst complex. Proc. Natl. Acad. Sci. USA *94*, 14438–14443.

Kurischko, C., Kuravi, V.K., Wannissorn, N., Nazarov, P.A., Husain, M., Zhang, C., Shokat, K.M., McCaffery, J.M., and Luca, F.C. (2008). The yeast LATS/Ndr kinase Cbk1 regulates growth via Golgi-dependent glycosylation and secretion. Mol. Biol. Cell *19*, 5559–5578.

Larsen, M.R., Thingholm, T.E., Jensen, O.N., Roepstorff, P., and Jørgensen, T.J. (2005). Highly selective enrichment of phosphorylated peptides from peptide mixtures using titanium dioxide microcolumns. Mol. Cell. Proteomics *4*, 873–886.

Lepore, D.M., Martínez-Núñez, L., and Munson, M. (2018). Exposing the elusive exocyst structure. Trends Biochem. Sci. *43*, 714–725.

Liu, J., Zuo, X., Yue, P., and Guo, W. (2007). Phosphatidylinositol 4,5-bisphosphate mediates the targeting of the exocyst to the plasma membrane for exocytosis in mammalian cells. Mol. Biol. Cell *18*, 4483–4492.

Macek, B., Carpy, A., Koch, A., Bicho, C.C., Borek, W.E., Hauf, S., and Sawin, K.E. (2017). Stable isotope labeling by amino acids in cell culture (SILAC) technology in fission yeast. Cold Spring Harb. Protoc. *2017*, top079814. PubMed.

Maeda, Y., Kashiwazaki, J., Shimoda, C., and Nakamura, T. (2009). The *Schizosaccharomyces pombe* syntaxin 1 homolog, Psy1, is essential in the development of the forespore membrane. Biosci. Biotechnol. Biochem. *73*, 339–345.

Martin, S.G., and Arkowitz, R.A. (2014). Cell polarization in budding and fission yeasts. FEMS Microbiol. Rev. *38*, 228–253.

Martin-Belmonte, F., and Perez-Moreno, M. (2011). Epithelial cell polarity, stem cells and cancer. Nat. Rev. Cancer *12*, 23–38.

Martín-Cuadrado, A.B., Dueñas, E., Sipiczki, M., Vázquez de Aldana, C.R., and del Rey, F. (2003). The endo-beta-1,3-glucanase eng1p is required for dissolution of the primary septum during cell separation in *Schizosaccharomyces pombe*. J. Cell Sci. *116*, 1689–1698.

Mayor, R., and Etienne-Manneville, S. (2016). The front and rear of collective cell migration. Nat. Rev. Mol. Cell Biol. *17*, 97–109.

Mazanka, E., Alexander, J., Yeh, B.J., Charoenpong, P., Lowery, D.M., Yaffe, M., and Weiss, E.L. (2008). The NDR/LATS family kinase Cbk1 directly controls transcriptional asymmetry. PLoS Biol. *6*, e203.

Mei, K., Li, Y., Wang, S., Shao, G., Wang, J., Ding, Y., Luo, G., Yue, P., Liu, J.J., Wang, X., et al. (2018). Cryo-EM structure of the exocyst complex. Nat. Struct. Mol. Biol. *25*, 139–146.

Millecamps, S., and Julien, J.P. (2013). Axonal transport deficits and neurodegenerative diseases. Nat. Rev. Neurosci. *14*, 161–176.

Mishra, M., Huang, J., and Balasubramanian, M.K. (2014). The yeast actin cytoskeleton. FEMS Microbiol. Rev. *38*, 213–227.

Mutavchiev, D.R., Leda, M., and Sawin, K.E. (2016). Remodeling of the fission yeast Cdc42 cell-polarity module via the Sty1 p38 stress-activated protein kinase pathway. Curr. Biol. *26*, 2921–2928.

Nakano, K., Toya, M., Yoneda, A., Asami, Y., Yamashita, A., Kamasawa, N., Osumi, M., and Yamamoto, M. (2011). Pob1 ensures cylindrical cell shape by coupling two distinct rho signaling events during secretory vesicle targeting. Traffic *12*, 726–739.

Nuñez, I., Rodriguez Pino, M., Wiley, D.J., Das, M.E., Chen, C., Goshima, T., Kume, K., Hirata, D., Toda, T., and Verde, F. (2016). Spatial control of translation repression and polarized growth by conserved NDR kinase Orb6 and RNA-binding protein Sts5. eLife *5*, e14216.

Olsen, J.V., Macek, B., Lange, O., Makarov, A., Horning, S., and Mann, M. (2007). Higher-energy C-trap dissociation for peptide modification analysis. Nat. Methods *4*, 709–712.

Ong, S.E., and Mann, M. (2006). A practical recipe for stable isotope labeling by amino acids in cell culture (SILAC). Nat. Protoc. *1*, 2650–2660.

Pearce, L.R., Komander, D., and Alessi, D.R. (2010). The nuts and bolts of AGC protein kinases. Nat. Rev. Mol. Cell Biol. *11*, 9–22.

Petersen, J., and Russell, P. (2016). Growth and the environment of *Schizosaccharomyces pombe*. Cold Spring Harb. Protoc. *2016*, top079764. PubMed.

Peterson, M.R., Burd, C.G., and Emr, S.D. (1999). Vac1p coordinates Rab and phosphatidylinositol 3-kinase signaling in Vps45p-dependent vesicle docking/fusion at the endosome. Curr. Biol. *9*, 159–162.

Racki, W.J., Bécam, A.M., Nasr, F., and Herbert, C.J. (2000). Cbk1p, a protein similar to the human myotonic dystrophy kinase, is essential for normal morphogenesis in *Saccharomyces cerevisiae*. EMBO J. *19*, 4524–4532.

Rappsilber, J., Ishihama, Y., and Mann, M. (2003). Stop and go extraction tips for matrix-assisted laser desorption/ionization, nanoelectrospray, and LC/MS sample pretreatment in proteomics. Anal. Chem. *75*, 663–670.

Ray, S., Kume, K., Gupta, S., Ge, W., Balasubramanian, M., Hirata, D., and McCollum, D. (2010). The mitosis-to-interphase transition is coordinated by cross talk between the SIN and MOR pathways in *Schizosaccharomyces pombe*. J. Cell Biol. *190*, 793–805.

Raychaudhuri, S., and Prinz, W.A. (2010). The diverse functions of oxysterol-binding proteins. Annu. Rev. Cell Dev. Biol. *26*, 157–177.

Rehberg, K., Kliche, S., Madencioglu, D.A., Thiere, M., Müller, B., Meineke, B.M., Freund, C., Budinger, E., and Stork, O. (2014). The serine/threonine kinase Ndr2 controls integrin trafficking and integrin-dependent neurite growth. J. Neurosci. *34*, 5342–5354.

Rodriguez-Boulan, E., and Macara, I.G. (2014). Organization and execution of the epithelial polarity programme. Nat. Rev. Mol. Cell Biol. *15*, 225–242.

Schelski, M., and Bradke, F. (2017). Neuronal polarization: From spatiotemporal signaling to cytoskeletal dynamics. Mol. Cell. Neurosci. *84*, 11–28.

Shevchenko, A., Wilm, M., Vorm, O., and Mann, M. (1996). Mass spectrometric sequencing of proteins silver-stained polyacrylamide gels. Anal. Chem. *68*, 850–858.

Simanis, V. (2015). Pombe's thirteen—control of fission yeast cell division by the septation initiation network. J. Cell Sci. *128*, 1465–1474.

Snaith, H.A., Thompson, J., Yates, J.R., 3rd, and Sawin, K.E. (2011). Characterization of Mug33 reveals complementary roles for actin cable-dependent transport and exocyst regulators in fission yeast exocytosis. J. Cell Sci. *124*, 2187–2199.

St Johnston, D., and Ahringer, J. (2010). Cell polarity in eggs and epithelia: parallels and diversity. Cell *141*, 757–774.

Tatebe, H., Nakano, K., Maximo, R., and Shiozaki, K. (2008). Pom1 DYRK regulates localization of the Rga4 GAP to ensure bipolar activation of Cdc42 in fission yeast. Curr. Biol. *18*, 322–330.

TerBush, D.R., and Novick, P. (1995). Sec6, Sec8, and Sec15 are components of a multisubunit complex, which localizes to small bud tips in *Saccharomyces cerevisiae*. J. Cell Biol. *130*, 299–312.

Ultanir, S.K., Hertz, N.T., Li, G., Ge, W.P., Burlingame, A.L., Pleasure, S.J., Shokat, K.M., Jan, L.Y., and Jan, Y.N. (2012). Chemical genetic identification of NDR1/2 kinase substrates AAK1 and Rabin8 uncovers their roles in dendrite arborization and spine development. Neuron *73*, 1127–1142.

Verde, F., Wiley, D.J., and Nurse, P. (1998). Fission yeast orb6, a ser/thr protein kinase related to mammalian rho kinase and myotonic dystrophy kinase, is required for maintenance of cell polarity and coordinates cell morphogenesis with the cell cycle. Proc. Natl. Acad. Sci. USA *95*, 7526–7531.

Vizcaíno, J.A., Csordas, A., del-Toro, N., Dianes, J.A., Griss, J., Lavidas, I., Mayer, G., Perez-Riverol, Y., Reisinger, F., Ternent, T., et al. (2016). 2016 update of the PRIDE database and its related tools. Nucleic Acids Res. *44* (*D1*), D447–D456.

Wang, H., Tang, X., Liu, J., Trautmann, S., Balasundaram, D., McCollum, D., and Balasubramanian, M.K. (2002). The multiprotein exocyst complex is essential for cell separation in *Schizosaccharomyces pombe*. Mol. Biol. Cell *13*, 515–529.

Weiss, E.L. (2012). Mitotic exit and separation of mother and daughter cells. Genetics *192*, 1165–1202.

Wiley, D.J., Marcus, S., D'urso, G., and Verde, F. (2003). Control of cell polarity in fission yeast by association of Orb6p kinase with the highly conserved protein methyltransferase Skb1p. J. Biol. Chem. *278*, 25256–25263.

Willet, A.H., McDonald, N.A., and Gould, K.L. (2015). Regulation of contractile ring formation and septation in *Schizosaccharomyces pombe*. Curr. Opin. Microbiol. *28*, 46–52.

Wright, J., Kahn, R.A., and Sztul, E. (2014). Regulating the large Sec7 ARF guanine nucleotide exchange factors: the when, where and how of activation. Cell. Mol. Life Sci. *71*, 3419–3438.

Wu, B., and Guo, W. (2015). The exocyst at a glance. J. Cell Sci. *128*, 2957–2964.

Wu, H., Rossi, G., and Brennwald, P. (2008). The ghost in the machine: small GTPases as spatial regulators of exocytosis. Trends Cell Biol. *18*, 397–404.

Yu, F.X., and Guan, K.L. (2013). The Hippo pathway: regulators and regulations. Genes Dev. *27*, 355–371.

Zhang, X., Orlando, K., He, B., Xi, F., Zhang, J., Zajac, A., and Guo, W. (2008). Membrane association and functional regulation of Sec3 by phospholipids and Cdc42. J. Cell Biol. *180*, 145–158.

Zhao, B., Wei, X., Li, W., Udan, R.S., Yang, Q., Kim, J., Xie, J., Ikenoue, T., Yu, J., Li, L., et al. (2007). Inactivation of YAP oncoprotein by the Hippo pathway is involved in cell contact inhibition and tissue growth control. Genes Dev. *21*, 2747–2761.

STAR★METHODS

KEY RESOURCES TABLE

REAGENT or RESOURCE	SOURCE	IDENTIFIER
Antibodies		
Affinity-purified Sheep anti-GFP antibody	Homemade lab stock	N/A
Bacterial and Virus Strains		
Escherichia coli BL21-CodonPlus(DE3)-RIL	Agilent Technologies	Cat# 230245
Chemicals, Peptides, and Recombinant Proteins		
$^{13}C_6$ –labeled L-arginine-HCl	Sigma-Aldrich	Cat# 643440
$^{13}C_6{}^{15}N_2$–labeled L-lysine-2HCl	Sigma-Aldrich	Cat# 608041
3-BrB-PP1	Toronto Research Chemicals	Cat# A602985
3-MB-PP1	Toronto Research Chemicals	Cat# A602960
1-NM-PP1	Toronto Research Chemicals	Cat# A603003
Hydroxyurea	Sigma-Aldrich	Cat# H8627
Latrunculin A	Alpha laboratories	Cat# 129-04361
Soybean lectin	Sigma-Aldrich	Cat# L1395
Formaldehyde solution	Sigma-Aldrich	Cat# F1268
Fluorescence Brightener 28	Sigma-Aldrich	Cat# F3543
Phosphatase substrate tablet	Sigma-Aldrich	Cat# S0942
Coomassie brilliant blue	Sigma-Aldrich	Cat# B0149
Iodoacetamide	Sigma-Aldrich	Cat# I1149
Trifluoroacetic acid	Sigma-Aldrich	Cat# 6508
Acetonitrile	Fisher Chemicals	Cat# A955-212
Titansphere	GL Sciences	Cat# 5020-75010
2,5-dihydrobenzoic acid	Sigma-Aldrich	Cat# 149357
Ammonium hydroxide	Sigma-Aldrich	Cat# 338818
Protein G Dynabeads	Thermo Fisher Scientific	Cat# 10003D
Glutathione-agarose	Sigma-Aldrich	Cat# G4510
FITC-dextran (average M_w = 500kDa)	Sigma-Aldrich	Cat# FD500S
Gibson assembly kit	New England Biolab	Cat# E2611S
Endopeptidase LysC	Waco Chemicals	Cat# 129-02541
Trypsin	Thermo Fisher Scientific	Cat# 90057
Deposited Data		
MS proteomics data have been deposited to ProteomeXchange Consortium via PRIDE	This paper	PXD009408
Experimental Models: Organisms/Strains		
Schizosaccharomyces pombe. See Table S3 for full strain list.	This paper	NCBI:txid4896
Oligonucleotides		
Primers for whole-plasmid PCR (pREP41X)- reverse: catatgatttaacaaagcgactataag	Eurofins	OKS2746
Primers for whole-plasmid PCR (pREP41X)- forward: ggatccccgggtaaaagg	Eurofins	OKS2749
Primers for amplifying Orb6- forward (fragment 1): cgctttgttaaatcatatggataagaatgattacttacactttgaac	Eurofins	OKS2747
Primers for amplifying Orb6- reverse (fragment 1): aaatcaccgcctggtaaaaactccgcaatcaagtaaagatacagtgaat	Eurofins	OKS2759
Primers for amplifying Orb6- forward (fragment 2): attcactgtatctttacttgattgcggagtttttaccaggcggtgattt	Eurofins	OKS2760

(Continued on next page)

Continued

REAGENT or RESOURCE	SOURCE	IDENTIFIER
Primers for amplifying Orb6- reverse (fragment 2): tttacccggggatccttacaatgctcctttcatc	Eurofins	OKS2748
Primers for amplifying Sec5 Open reading frame- forward: tccaggggcccgcggaattcatgagtgcagacgaagagatcc	Eurofins	OKS3613
Primers for amplifying Sec5 Open reading frame- reverse: ggccgctcgagtcgacccggttatgaaaagatcatttcaataaactgaaacttg	Eurofins	OKS3616
Primers for amplifying Sec3 Open reading frame- forward: tccaggggcccgcggaattcatggcttctaaccctaatgcc	Eurofins	OKS3610
Primers for amplifying Sec3 Open reading frame- reverse: ggccgctcgagtcgacccggtcatctacggcttggttgtt	Eurofins	OKS3611
Recombinant DNA		
pREP41X-orb6-as2 plasmid	This paper	pKS1439
pREP41X *S. pombe* overexpression vector	Paul Nurse Lab, Francis Crick Institute	pKS2
Plasmid for expression of recombinant GST-Sec3	This paper	pKS1701
Plasmid for expression of recombinant GST-Sec5	This paper	pKS1702
pGEX4T-1 vector for recombinant protein expression	GE Healthcare	Cat# 28954549
Software and Algorithms		
Metamorph software	Molecular Devices	RRID:SCR_002368
ImageJ (FIJI)	NIH	RRID:SCR_002285
StackReg plugin	EPFL (Lausanne)	http://bigwww.epfl.ch/thevenaz/stackreg/
Reslice plugin	NIH	N/A
KymoResliceWide plugin	Eugene Katrukha (Utrecht)	https://imagej.net/KymoResliceWide
Photoshop	Adobe	RRID:SCR_014199
Illustrator CS3	Adobe	RRID:SCR_010279
Illustrator CS6	Adobe	RRID:SCR_010279
QuickTime	Apple	N/A
Graphpad Prism 7 software	Graphpad	RRID:SCR_002798
Microsoft Excel	Microsoft	RRID:SCR_016137
MaxQuant software platform version 1.5.2.8	Cox and Mann, 2008	RRID:SCR_014485
Pombe database (released in August, 2013)	Pombase	RRID:SCR_006586
Andromeda search engine	Cox et al., 2011	https://omictools.com/andromeda-tool
MegAlignPro version 15	DNASTAR	RRID:SCR_000291
Generic Gene Ontology (GO) Term Finder	Princeton University	RRID:SCR_008870
Other		
Freezer/mill LLC 6870	SPEX SamplePrep	N/A
Bolt 4-12% Bis-Tris Gel	Thermo Fisher Scientific	Cat# NW04120BOX
Resource S SCX column 1 mL	GE Healthcare	Cat# GE17-1178-01
ÄKTA protein purification system	GE Healthcare	N/A
Vacuum centrifugation concentrator 5301	Eppendorf	N/A
Q Exactive mass spectrometer	Thermo Fisher Scientific	N/A
Orbitrap Fusion Lumos Tribrid Mass Spectrometer	Thermo Fisher Scientific	N/A
Ultimate 3000 RSLCnano Systems Dionex	Thermo Fisher Scientific	N/A
Air-pressure pump	Proxeon Biosystems	N/A
Column oven	Sonnation	N/A
50cm EASY-Spray column	Thermo Fisher Scientific	N/A
Amicon Ultra-4 centrifugal filter unit with Ultracel-30 membrane	Millipore	Cat# UFC803024

(*Continued on next page*)

Continued		
REAGENT or RESOURCE	SOURCE	IDENTIFIER
Ribolyser	Hybaid	N/A
Coverslip dishes	MatTek	Cat# P35G-0.170-14-C.s
4-chamber glass bottom micro-slides	Ibidi	Cat# 80427

CONTACT FOR REAGENT AND RESOURCE SHARING

Further requests for reagents and resource sharing should be directed to and will be fulfilled by the Lead Contact, Prof. Kenneth Sawin (ken.sawin@ed.ac.uk).

EXPERIMENTAL MODEL AND SUBJECT DETAILS

Fission Yeast Strains and Construction

Mating for genetic crosses (Ekwall and Thon, 2017) was performed on SPA5S plates with supplements at 45 mg/L. Crosses were performed using tetrad dissection or random spore analysis. Tagging and deletion of genes were performed using PCR-based methods (Bähler et al., 1998). The *orb6-as2* strain was generated by transformation of an NdeI-BamHI restriction fragment containing the *orb6-as2* coding sequence (from pKS1439; see below) into an *orb6-25* strain. Selection was performed at 37°C, which is non-permissive for growth of *orb6-25* cells. Positive clones were sequenced to ensure correct replacement of the *orb6-25* allele by *orb6-as2*. All strains used in this study are listed in Table S3. Throughout this work, we use the term "wild-type" to refer to control strains that have wild-type alleles relative to the mutants with which they are compared in the same figure or panel. Therefore, strains described as "wild-type" can have additional auxotrophies and/or fluorescent-reporter alleles; full genotypes are indicated in strain list.

METHODS DETAILS

Yeast culture

Standard fission yeast methods were used throughout (Forsburg and Rhind, 2006; Petersen and Russell, 2016). Cultures for live-cell imaging and *in vitro* kinase assays were grown in YE5S rich medium (using Bacto yeast extract; Becton Dickinson). SILAC cultures were grown in Edinburgh Minimal Medium with appropriate supplements. Supplements such as adenine, leucine, and uracil were used at 175 mg/L. Solid media used 2% Bacto agar (Becton Dickinson). Normal arginine and lysine supplements were used at 40 mg/L and 30 mg/L, respectively. "Heavy" $^{13}C_6$ –labeled L-arginine-HCl ($^{13}C_6$ –labeled; Sigma; 643440) and $^{13}C_6$ $^{15}N_2$ –labeled L-lysine-2HCl ($^{13}C_6$ $^{15}N_2$–labeled; Sigma; 608041) supplements were used at the same concentration as their light counterparts. SILAC cell cultures were grown as described previously (Bicho et al., 2010; Macek et al., 2017). To inhibit Orb6 kinase activity *in vivo*, 3-BrB-PP1 (Toronto Research Chemicals; A602985), 3-MB-PP1 (Toronto Research Chemicals; A602960), 1-NM-PP1 (Toronto Research Chemicals; A603003) dissolved in methanol were each used at a final concentration of 30 μM. 3-MB-PP1 and 1-NM-PP1 were only sparingly soluble in methanol in normal stock solutions, whereas 3-BrB-PP1 was considerably more soluble; therefore we used 3-BrB-PP1 for Orb6 inhibition in all other experiments. For imaging experiments involving hydroxyurea, cells were grown in YE5S media containing 12 mM hydroxyurea (prepared fresh; Sigma; H8627), starting at 1.4 hr before the beginning of imaging. For actin depolymerization, Latrunculin A (Alpha laboratories; 129-04361) was added to cells at a final concentration of 50 μM in growth medium (made from a 25 mM stock in DMSO), 10 min before addition of 3-BrB-PP1.

Plasmid construction

Two DNA fragments encoding N- and C-terminal regions of Orb6 were amplified by PCR from fission yeast genomic DNA. Both DNA sequences contain a 25-nucleotide overlapping sequence and a single point mutation (*orb6-as2*) corresponding to an M170A mutation in the amino-acid sequence. The two fragments were assembled with plasmid pREP41X using Gibson assembly kit (NEB; E2611S), to make plasmid pKS1439. Sec3 and Sec5 sequences amplified from fission yeast genomic DNA were cloned into pGEX4T-1 vector and expressed as GST fusion proteins in *E.coli* strain BL21(DE3)-RIL (Agilent).

Microscopy sample preparation and imaging

Exponentially growing cells cultured at 25°C were used in all imaging experiments, unless otherwise stated. Fluorescence live-cell imaging was performed either in coverslip dishes (MatTek; P35G-0.170-14-C.s) or 4-chamber glass bottom micro-slides (Ibidi; 80427). Imaging dishes/slides were coated with 1 mg/mL soybean lectin (Sigma; L1395) for 10 min and washed with appropriate medium to remove excess lectin. Log-phase culture was added to dishes/slides and allowed to attach to the coverslip bottom for 15 min. The dishes/slides were washed extensively with media using aspiration with at least 3 full exchanges of media (approximately 1 mL each). Finally, 500 μL of medium was added to the dish/slide before imaging. Nearly all live-cell fluorescence imaging was done using

a custom spinning-disk confocal microscope unit composed of Nikon TE2000 microscope base, attached to a modified Yokogawa CSU-10 unit (Visitech) and an iXon+ Du888 EMCCD camera (Andor), 100x/1.45 NA Plan Apo objective (Nikon), Optospin IV filter wheel (Cairn Research), MS-2000 automated stage with CRISP autofocus (ASI), and thermo-regulated chamber maintained at 25°C (OKOlab). Metamorph software was used to control the spinning-disc confocal microscope. FRAP was performed on a Zeiss Airyscan microscope (LSM880, AxioObserver, alpha Plan-ApoChromat 100x/1.46 Oil DIC M27 Elyra). GFP signal was detected using 488 nm line of an Argon laser and a 495-550 nm bandpass emission filter (number of iterations = 5, acquisition time interval 30 s). To measure width of live cells attached to coverslip dishes, FITC-dextran (Sigma: FD500S) was added to the medium at final concentration of 3.5mg/mL and imaged using 488nm excitation laser.

Calcofluor staining was performed according to standard protocol (Hagan, 2016). Briefly, 900 μL of log-phase culture was added to 100 μL 30% formaldehyde solution (Sigma; F1268) and kept on ice for 10 min. Cells were pelleted and washed three times using ice-cold PBS buffer. The final pellet was resuspended in 50 μL PBS. 1.5μL of cell suspension was mixed with 1.5 μL of 10 μg/mL Calcofluor solution (Fluorescence Brightener 28; Sigma; F3543). Calcofluor-stained cells were imaged using a Zeiss AxioImage microscope with 100X/1.46NA alpha-Plan Apochromat objective, CoolLED pE-300 lightsource and Hamamatsu Flash 4 camera. Micromanager acquisition software was used to control the Zeiss microscope.

For imaging experiments, we define a "biological replicate" as the collective imaging of all relevant, validated strains on a single occasion under the specific conditions stated. In a given (replicate) imaging experiment for a given strain, multiple independent fields were imaged, with multiple cells per field. For presentation in figures, representative cells were chosen.

Microscopy image analysis

ImageJ (Fiji) was used to process all acquired raw images. All images and videos shown are maximum projections of eleven Z sections with 0.7 μm step-size. ImageJ StackReg plugin was used for rigid body registrations. Kymographs was generated using Reslice and KymoResliceWide plugin in ImageJ. Cell-width and cell-elongation measurements were performed manually using the Line tool in ImageJ. Cell volume was estimated using the measured length and width of individual cells, with the approximation that each cell can be considered as a cylinder plus one hemisphere at each end. FRAP of GFP-Psy1 was quantified by measuring total fluorescence signal within the bleached portion of the plasma membrane (orange portion of linescan in Figure 3C) and normalizing values to the signal for the same region prior to bleaching. Image formatting and assembly were performed using Photoshop (Adobe) and Illustrator CS3 or CS6 (Adobe). Videos were edited using ImageJ and QuickTime (Apple). Graphs were created using GraphPad Prism software and Microsoft Excel.

ACP assay

ACP assays were performed as described previously (Wang et al., 2002). Cells were grown to mid-log phase in YE5S at 25°C. Cells were washed twice in pre-warmed YE5S medium, and all strains were adjusted to OD_{595} = 0.25. The cells in fresh media were cultured in a 25°C shaking water bath. At indicated time points, cell density (OD_{595}) was measured, and 1 mL culture was centrifuged to pellet the cells. 300 μL cell-free culture medium was incubated with 400 μL of phosphatase substrate solution (3 tablets of phosphatase substrate (Sigma; S0942) dissolved in 20 mL of 0.1 M sodium acetate pH 4.0, pre-warmed to 32°C). The reaction was stopped by addition of 400 μL of 1 M NaOH. OD_{405} was measured against a blank consisting of fresh YE5S medium without cells, mixed with an appropriate volume of phosphatase substrate solution. For each time point, ACP activity (OD_{405}) was normalized to cell density (OD_{595}). In the experiments in Figure 3B, four independent biological replicates were performed for wild-type and *orb6-as2*, and two independent biological replicates for *orb6-as2 gef1Δ*. In the experiments in Figure 7A, three independent replicates were performed for all strains.

Sample preparation for global phosphoproteomics

SILAC cultures supplemented with "heavy" arginine and lysine were grown for at least 8 generations to ensure complete isotopic labeling. Light- and heavy-labeled SILAC cultures were grown to OD_{595} 0.75 and then treated with either methanol or 30 μM 3-BrB-PP1 for 3 hr. In two replicate experiments, heavy-labeled cultures were treated with 3-BrB-PP1, and light-labeled cultures were treated with methanol; in a third replicate, heavy-labeled cultures were treated with methanol, and light-labeled cultures were treated with 3-BrB-PP1. Cells were harvested by centrifugation and washed once with STOP buffer (10 mM EDTA, 50 mM NaF, 150 mM NaCl, 1 mM NaN_3). Pelleted cells were resuspended in milliQ H_2O at 400 mg/mL concentration and flash-frozen in liquid nitrogen. Mechanical lysis of frozen cells (cryogrinding) was performed under liquid nitrogen in a SPEX SamplePrep LLC 6870 Freezer/mill® (sample pre-cool for 2 min, 10 rounds of "run" and "cool" cycle each for 2 min, beat rate = 10). ~800 mg of light- and heavy-labeled cell powders were individually solubilized in denaturation buffer (6 M urea, 2 M thiourea, 10 mM Tris-HCl pH 8.0) for 1 hr at room temperature and centrifuged at 4,500 x *g* for 15 min to obtain a clear supernatant. Protein concentration from both samples was measured using Bradford assay.

For protein abundance measurement of the SILAC samples, light- and heavy-labeled cell lysates (5 mg each) were mixed, and 50 μg of the mixed protein lysate was separated by SDS-PAGE on Bolt® 4%–12% Bis-Tris Gel (Thermo Fisher Scientific; NW04120BOX). The protein gel was stained by Coomassie brilliant blue and the entire gel lane was excised into 10 bands and processed for protein abundance analysis (see In-Gel Digestion for Mass Spectrometry).

For SILAC phosphopeptide quantification, the remaining 9.95 mg of the mixed protein lysate was reduced in 1 mM DTT for 1 hr at room temperature. The reduced lysate was further alkylated in 5.5 mM iodoacetamide (Sigma; I1149) for 1 hr at room temperature in the dark. In-solution protein digestions were performed using Lysyl Endopeptidase (LysC; Waco Chemicals; 129-02541) and trypsin (Thermo-scientific; 90057) in two stages. First, the lysate was digested by 90 μg of LysC for 3 hr at room temperature. The lysate was then diluted 5 times to final concentration of 1.6 M urea/ 0.4 M thiourea. 90 μg of trypsin was added to the lysate and incubated for 24 hr at room temperature. Digestion was terminated by acidification to final concentration of 0.4% trifluoroacetic acid (TFA; Sigma; 6508).

Peptide fractionation

Strong cation exchange (SCX) fractionation was performed as in (Gruhler et al., 2005) with some modifications. Doubly digested mixed lysate was fractionated using a Resource S SCX column (1 mL, GE Healthcare) in a ÄKTA protein purification system (GE Healthcare). Briefly, the mixed lysate was loaded onto the SCX column equilibrated in Solvent A (5 mM potassium dihydrogen phosphate, 30% acetonitrile (ACN; Fisher Chemicals, A955-212), pH 2.7 with TFA) at a flow rate of 1 mL/min. Flow-through during loading was kept for subsequent phosphopeptide enrichment. Peptides bound to the column were eluted as 2 mL fractions with a 0%–50% linear gradient of Solvent B (350 mM potassium chloride, 5 mM potassium dihydrogen phosphate, 30% ACN, pH 2.7 using TFA) at a flow rate of 1 mL/ min, over a period of 30 min. Dilute fractions, based on estimation from the chromatogram, were pooled together for subsequent phosphopeptide enrichment.

Phosphopeptide enrichment

TiO_2 phosphopeptide enrichment was performed as in (Larsen et al., 2005) with some modification. Each SCX fraction, including the flow-through and pooled fractions, was processed individually. Flow-through fraction was incubated with 10 mg TiO_2 beads slurry (Titansphere; GL Sciences; 5020-75010) in 30 mg/mL 2,5 dihydrobenzoic acid (Sigma; 149357) and 80% ACN, whereas other fractions were incubated with 5 mg TiO_2 beads slurry and incubated at room temperature for 30 min. TiO_2 beads were concentrated by 1 min centrifugation at 13,000 rpm and supernatant was removed. TiO_2 beads were then washed with washing solution I (30% ACN, 3% TFA) and pelleted. TiO_2 beads were then resuspended with washing solution II (80%, ACN, 0.1% TFA) and transferred to C8-stage tip (~1 mm^2 Empore C8 in 200 μL pipette tip). Washing solution II was removed from the stage-tip by centrifugation at 4,000rpm for 2-3 min. Phosphopeptides bound to the TiO_2 beads were eluted three times by passing through elution solution (40% ammonium hydroxide (Sigma; 338818), 60% ACN). The eluates were loaded onto C18-stage tip (~1mm^2 Empore C18 in 200 μL pipette tip) pre-washed with 0.1% TFA. The stage tips were washed with 50 μL 0.1% TFA and stored at −20°C until further use. Because the flow-through fraction contains the most abundant phosphopeptide fraction, two additional rounds of phosphopeptide enrichment were performed on the flow-through fraction with fresh TiO_2 beads (10 mg).

In-gel digestion for MS

Coomassie-stained gel slices were destained and digested in-gel as described (Shevchenko et al., 1996). Briefly, the bands were destained by 3-4 rounds of incubation in 25 mM ammonium bicarbonate in 50% ACN until the last trace of Coomassie blue was removed. Gel slices were reduced in 10 mM DTT for 30 min at 37°C, alkylated in 55 mM iodoacetamide for 30 min at room temperature in the dark, and digested overnight at 37°C with 12.5 ng/μL trypsin. Digestion medium was then acidified by TFA and the eluted peptides were desalted using C18-stage tip as described (Rappsilber et al., 2003).

LC-MS/MS

Peptides were eluted in 40 μL of 80% acetonitrile in 0.1% TFA and then concentrated to 1 μL by vacuum centrifugation (Concentrator 5301, Eppendorf, UK). Samples were then diluted to 5 μL with 0.1% TFA and prepared for LC-MS/MS analysis. LC-MS-analyses were performed on a Q Exactive mass spectrometer (Thermo Fisher Scientific, UK) and on an Orbitrap Fusion Lumos Tribrid Mass Spectrometer (Thermo Fisher Scientific, UK). Both were coupled on-line to Ultimate 3000 RSLCnano Systems (Dionex, Thermo Fisher Scientific, UK). For samples that were analyzed by Q Exactive, peptides were separated by an analytical column with a self-assembled particle frit (Ishihama et al., 2002) and C18 material (ReproSil-Pur C18-AQ 3 μm; Dr. Maisch, GmbH, Germany) that was packed into a spray emitter (75 μm ID, 8 μm opening, 300 mm length; New Objective) using an air-pressure pump (Proxeon Biosystems, USA). The column was maintained at stable temperature (40°C) with the appropriate column oven (Sonnation, Germany). Peptides that were analyzed by Fusion Lumos were separated on a 50cm EASY-Spray column (Thermo Fisher Scientific, UK) assembled in an EASY-Spray source and operated at 50°C. In both cases, mobile phase A consisted of 0.1% formic acid in water while mobile phase B consisted of 80% acetonitrile and 0.1% formic acid. Peptides were loaded at a flow rate of 0.5 μL min^{-1} (for samples on Q Exactive) and 0.3 μL min^{-1} (for samples on Fusion Lumos) and eluted at a flow rate was 0.2 μL min^{-1}according to the following gradient: 2 to 40% buffer B in 120 min, then to 95% in 11 min.

For Q Exactive, FTMS spectra were recorded at 70,000 resolution (scan range 300-1700 m/z), and the ten most intense peaks with charge ≥ 2 of the MS scan were selected with an isolation window of 2.0 Thomson for MS2 (filling 1.0E6 ions for MS scan, 5.0E4 ions for MS2, maximum fill time 60ms, dynamic exclusion for 50 s). For Orbitrap Fusion Lumos, survey scans were performed at 120,000 resolution (scan range 400-1900 m/z) with an ion target of 4.0e5. MS2 was performed in the ion trap with ion target of 1.0e4 and HCD fragmentation with normalized collision energy of 27 (Olsen et al., 2007). The isolation window in the quadrupole was 1.4. Only ions with charge between 2 and 7 were selected for MS2.

The MaxQuant software platform version 1.5.2.8 was used to process raw files, and search was conducted against *Schizosaccharomyces pombe* complete/reference proteome set of Pombe database (released in August, 2013), using the Andromeda search engine. The first search peptide tolerance was set to 20 ppm while the main search peptide tolerance was set to 4.5 ppm. Isotope mass tolerance was 2 ppm and maximum charge to 7. Maximum of two missed cleavages were allowed. Carbamidomethylation of cysteine was set as fixed modification. Oxidation of methionine, acetylation of the N-terminal and phosphorylation of serine, threonine and tyrosine were selected as variable modifications. When SILAC labeled samples were analyzed, the multiplicity was set to 2 and the appropriate labels were selected. Peptide and protein identifications were filtered to 1% FDR.

Global phosphoproteomics data analysis and presentation

For summaries of global phosphoproteomics results, presented in Table S1, selected MaxQuant fields from individual biological-replicate experiments are shown in separate tabs (full datasets containing all fields are available via the PRIDE repository; see below). To generate the ''Orb6 inhibition summary'' tab, only phosphosites with localization probability greater than or equal to 0.75 from a given replicate experiment were copied into the summary tab. This decreased the number of quantified phosphosites after Orb6 inhibition from 8,134 to 6,300. In the summary tab, the mean log_2(fold-change) in phosphorylation of a given phosphosite after Orb6 inhibition was calculated in one of two ways, depending on whether the log_2(fold-change) values in a replicate experiment included normalization to protein abundance. For phosphosites with values normalized to protein abundance, only the protein-normalized values were used to calculate mean log_2(fold-change). That is, non-protein-normalized values for the same phosphosite were not used in the calculation, even though these values are listed in the summary tab. For phosphosites lacking any log_2(fold-change) values normalized to protein abundance, the non-protein-normalized values were used in the calculation. The mean of the log_2(fold-change) was used rather than the log_2(mean fold-change) in order to decrease effects of outlier data.

For ranking of Orb6-dependent phosphosites (based on fold-change in phosphorylation after Orb6 inhibition) in relation to the NDR/LATS consensus motif, presented in Table S2, different ''stringency'' tabs were made in order to take into consideration the reproducibility of phosphosite quantification in different biological-replicate experiments. To populate the different tabs in Table S2, data from the ''Orb6_inhibition summary'' tab in Table S1 were used as initial input. In order for a given phosphosite to be included in the ''Lowest Stringency'' tab, three conditions had to be satisfied. First, the phosphosite was required to be quantified in at least one of three biological-replicate experiments. Second, the phosphosite was required to have a greater-than-two-fold decrease after Orb6 inhibition (i.e., the ''mean log_2(fold-change) after Orb6 inhibition'' was required to be more negative than -1.0). Third, the same phosphosite was required not to have a greater-than-two-fold decrease in the corresponding control experiment in which wild-type cells were treated with 3-BrB-PP1 (i.e., the ''mean log_2(fold-change) after 3-BrB-PP1 addition'' in the Control tab in Table S1 was not allowed to be more negative than -1.0). In order for a given phosphosite to be included in the ''Medium Stringency'' tab, the previous latter two conditions also applied, but the phosphosite was required to be quantified in at least two biological replicates. Similarly, in order for a given phosphosite to be included in the ''Highest Stringency'' tab, the same latter two conditions also applied, but the phosphosite was required to be quantified in all three biological replicates. Based on these conditions, all phosphosites in the ''Highest Stringency'' tab are also present in the ''Medium Stringency'' tab, and all phosphosites in the ''Medium Stringency'' tab are also present in the ''Lowest Stringency'' tab. Within each tab, phosphosites were then ranked by fold-decrease after Orb6 inhibition, and then classified according to similarity to the NDR/LATS consensus motif, HxR/H/KxxS/T. Alignment of sequences containing the NDR/LATS consensus was performed using MegAlignPro version 15 (DNASTAR).

GO analysis for Figure 4C was carried out using Generic Gene Ontology (GO) Term Finder. Fold-enrichment for each GO term identified from our input cluster of genes is defined as the ratio of (frequency of the GO term within the cluster) to (frequency of the GO term within the genome).

Immunoprecipitation of Sec5-3mCherry

Cells expressing Sec5-3mCherry in *SILAC* background were grown in two separate cultures supplemented with light and heavy isotopes for 8 generations. Light- and heavy-labeled cultures were treated with methanol and 30 μM 3-BrB-PP1 respectively, for 45 min. The cells were washed with STOP buffer and resuspended in lysis buffer (50 mM Tris-HCl pH 7.5, 50 mM NaF, 150 mM NaCl, 20 mM Na-β-glycerophosphate, 0.2% Triton X-100, 1 mM Na_3VO_4, 1 mM EDTA, 10 μg/mL each of 'CLAAPE' protease inhibitors (chymostatin, leupeptin, aprotinin, antipain, pepstatin, E-64), 2 mM AEBSF, 2 mM benzamidine, 50 nM Calyculin A, 50 nM Okadaic acid and 2 mM PMSF) at a concentration of 1 g/mL. The cell suspension was drop-frozen in liquid nitrogen and mechanically lysed using SPEX Freezer/Mill®. Light- and heavy-labeled cell powders were resuspended in lysis buffer and cell extracts were cleared by 2 × 15 min centrifugation at 4,000 x *g* in a Beckman Avanti J-26 centrifuge using a JLA 8.1000 rotor. Protein content in each sample was measured and samples are mixed at 1:1 ratio. The mixed cell extract was incubated with Protein G Dynabeads (Thermo Fisher Scientific; 10003D), covalently crosslinked to homemade affinity-purified rabbit anti-tdimer2 using dimethyl pimelimidate, for 1 hr. Dynabeads were then washed five times with lysis buffer. Proteins were eluted from Dynabeads by 10 min incubation at 65°C in Laemmli sample buffer. Eluted proteins were separated by SDS-PAGE on a Bolt® 4%–12% Bis-Tris Gel. The gel was stained by Coomassie Brilliant Blue, and gel bands were excised and processed according to the in-gel digestion protocol (see above).

Purification of GST-Sec3 and GST-Sec5

Expression was induced in 500 mL bacterial culture at OD_{595} = 0.8 by addition of 0.2 mM IPTG, followed by growth for additional 24 hr at 18°C. Cells were harvested by centrifugation and resuspended in lysis buffer (50 mM Tris-HCl pH 8.0, 150 mM NaCl, 5% glycerol, 5 mM EDTA, 3 mM DTT, 0.1% Triton X-100, 200 μg/mL lysozyme, benzonase (10 units/mL), 10 μg/mL each of 'CLAAPE' protease inhibitors). The cells were then lysed by sonication. Cell extracts were clarified by centrifugation at 15,000 rpm at 4°C for 30 min. Cleared lysates were then incubated with glutathione-agarose (Sigma; G4510). Glutathione-agarose with bound GST proteins was then placed in a column and washed with 20 bead-volumes of wash buffer (50 mM Tris-HCl pH 8.0, 300 mM NaCl, 100 mM KCl, 10% glycerol, 5 mM EDTA, 3 mM DTT, 0.1% Triton X-100). GST fusion proteins were then eluted with elution buffer (50 mM Tris-HCl pH 8.0, 300 mM NaCl, 10% glycerol, 2 mM EDTA, 3 mM DTT, 20 mM glutathione) in 1 mL fractions. Protein-rich fractions were pooled together and concentrated using Amicon Ultra-4 centrifugal filter unit with Ultracel®-30 membrane (Millipore; UFC803024).

In vitro kinase assays

Orb6 *in vitro* kinase assays were performed using immunoprecipitates of the Orb6 coactivator Mob2, as immunoprecipitation using antibody bound to Orb6 can inhibit kinase activity (Wiley et al., 2003). Cells expressing Mob2-GFP in *sts5Δ* and *sts5Δ orb6Δ* backgrounds were grown in YE5S medium to mid-log-phase, harvested and washed with STOP buffer. $1x10^7$ Cells were pelleted into 1.5 mL screw-cap microfuge tubes and snap frozen in liquid nitrogen. 200 μL of precooled lysis buffer (50 mM Tris-HCl pH 7.5, 50 mM NaF, 150 mM NaCl, 20 mM Na-β-glycerophosphate, 0.2% Triton X-100, 1 mM Na_3VO_4, 1 mM EDTA, 10 μg/mL each of 'CLAAPE' protease inhibitors, 2 mM AEBSF, 2 mM benzamidine, 50 nM calyculin A, 50 nM okadaic acid and 2 mM PMSF) and ~500 μL of 0.5 mm zirconia beads were added to the microfuge tubes and disrupted by bead beating in Ribolyser (Hybaid) at 4°C using two cycles of 35 s at maximum setting (6.5). 350 μL of lysis buffer was added into the tube and mixed thoroughly. Cell lysates were collected by centrifugation through a hole pierced at the bottom of the microfuge tube. Cell lysates were cleared by centrifugation at 13,000 rpm for 6 min at 4°C. 550 μL of cell lysates were incubated with 10 μL of Protein G Dynabeads, coupled to homemade affinity-purified Sheep anti-GFP, for 1 hr at 4°C. Dynabeads were then washed 3 times using lysis buffer and 3 times using kinase buffer (50 mM Tris-HCl pH 7.5, 100 mM NaCl, 10 mM $MgCl_2$, 1 mM $MnCl_2$, 10 mM ATP, 20 mM Na-β-glycerophosphate, 1 mM Na_3VO_4, 10 μg/mL each of 'CLAAPE' protease inhibitors). Dynabeads were then incubated with 5 μL of recombinant GST-Sec3 and GST-Sec5 in 20 μL kinase buffer at 30°C for 30 min. The kinase reaction was stopped and recovered from Dynabeads by incubation in Laemmli sample buffer at 65°C for 10 min. Kinase reactions and controls were separated on Bolt® 4%–12% Bis-Tris gel and stained with Coomassie. Bands corresponding to GST-Sec3 and GST-Sec5 were excised and processed according to in-gel digestion protocol.

QUANTIFICATION AND STATISTICAL ANALYSIS

Statistical tests

Statistical analyses in Figures 1C and 1E–1G were determined by two-tailed unpaired Mann–Whitney test, using GraphPad Prism software (see Microscopy Image Analysis). Statistical significance in Figure 4C was determined by Generic Gene Ontology (GO) Term Finder (see phosphosite ranking and GO analysis as described earlier).

DATA AND SOFTWARE AVAILABILITY

The MS proteomics data have been deposited to the ProteomeXchange Consortium via the PRIDE (Vizcaíno et al., 2016) partner repository with the dataset identifier PXD009408.

Cell Reports, Volume *26*

Supplemental Information

Fission Yeast NDR/LATS Kinase Orb6 Regulates Exocytosis via Phosphorylation of the Exocyst Complex

Ye Dee Tay, Marcin Leda, Christos Spanos, Juri Rappsilber, Andrew B. Goryachev, and Kenneth E. Sawin

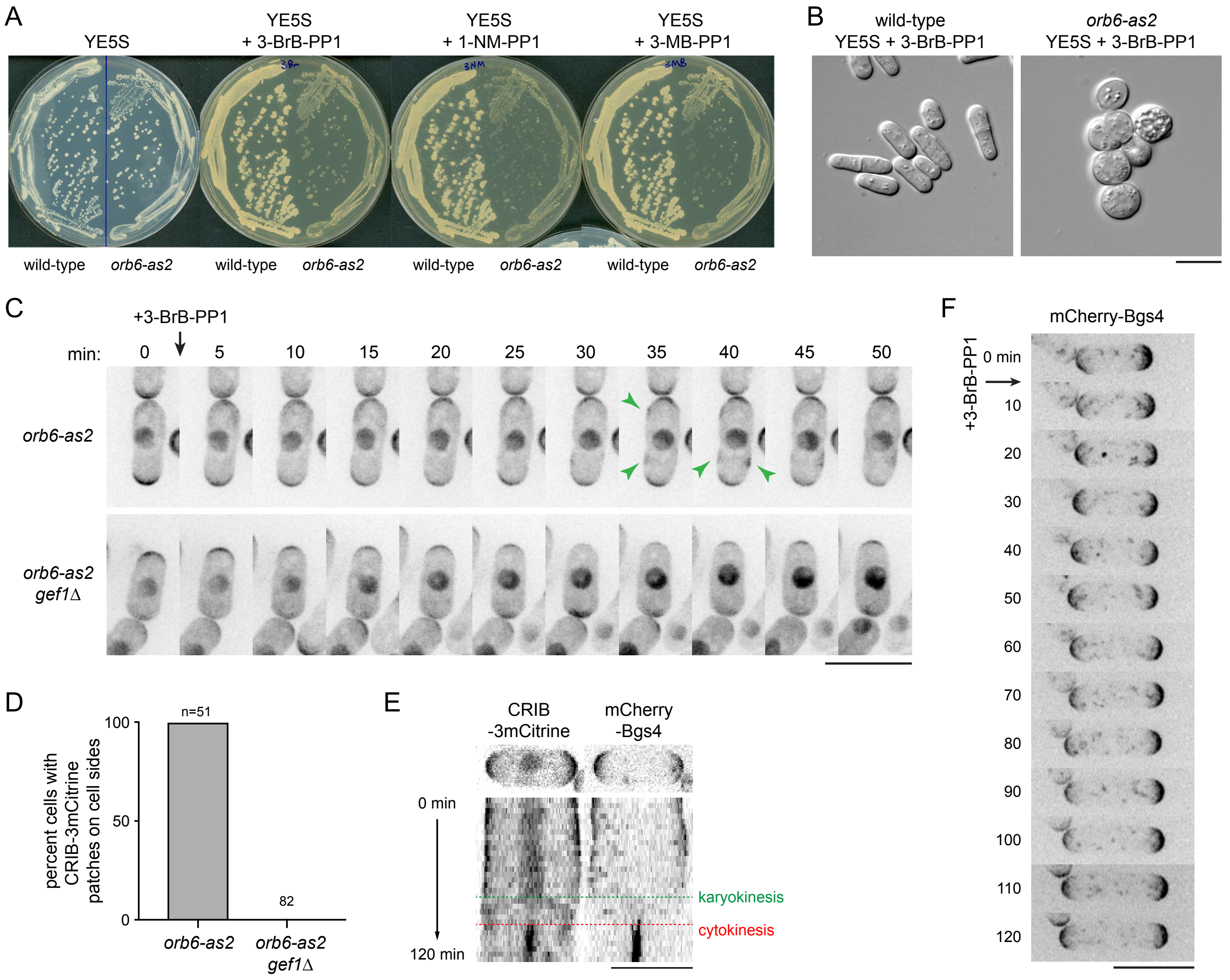

Figure S1. Additional characterization of *orb6-as2* and effects of 3-BrB-PP1. Related to Figures 1 and 2.
(A) Wild-type and *orb6-as2* colonies after replica-plating to the different indicated nucleotide-competitive analogs (each analog at 30 μM final concentration). All analogs inhibited growth of *orb6-as2* cells. Plates were incubated at 32ºC for 2 days.
(B) DIC images of wild-type and *orb6-as2* cells taken from YE5S + 3-BrB-PP1 plate. On solid media at 32°C, Orb6-inhibited cells are rounder than in liquid medium at 25°C; this is partly due to elevated temperature (see Figure 1).
(C) CRIB-3mCitrine (Cdc42-GTP reporter) localization in interphase *orb6-as2* and *orb6-as2 gef1*Δ cells before and after Orb6 inhibition with 3-BrB-PP1. After Orb6 inhibition, ectopic patches of CRIB-3mCitrine are observed in *orb6-as2* cells (arrowheads) but not in *orb6-as2 gef1*Δ cells.
(D) Quantification of ectopic CRIB-3mCitrine patches from experiments as in (C).
(E) Temporal correlation of CRIB-3mCitrine and mCherry-Bgs4 localization to cell tips during interphase and to the division site during cell division.
(F) Addition of 3-BrB-PP1 (30 μM) to wild-type cells does not affect mCherry-Bgs4 localization or interphase cell elongation.
Bars, 10 μm. Three biological replicates were performed for (C) and (E). Experiments for (A), (B) and (F) were performed once.

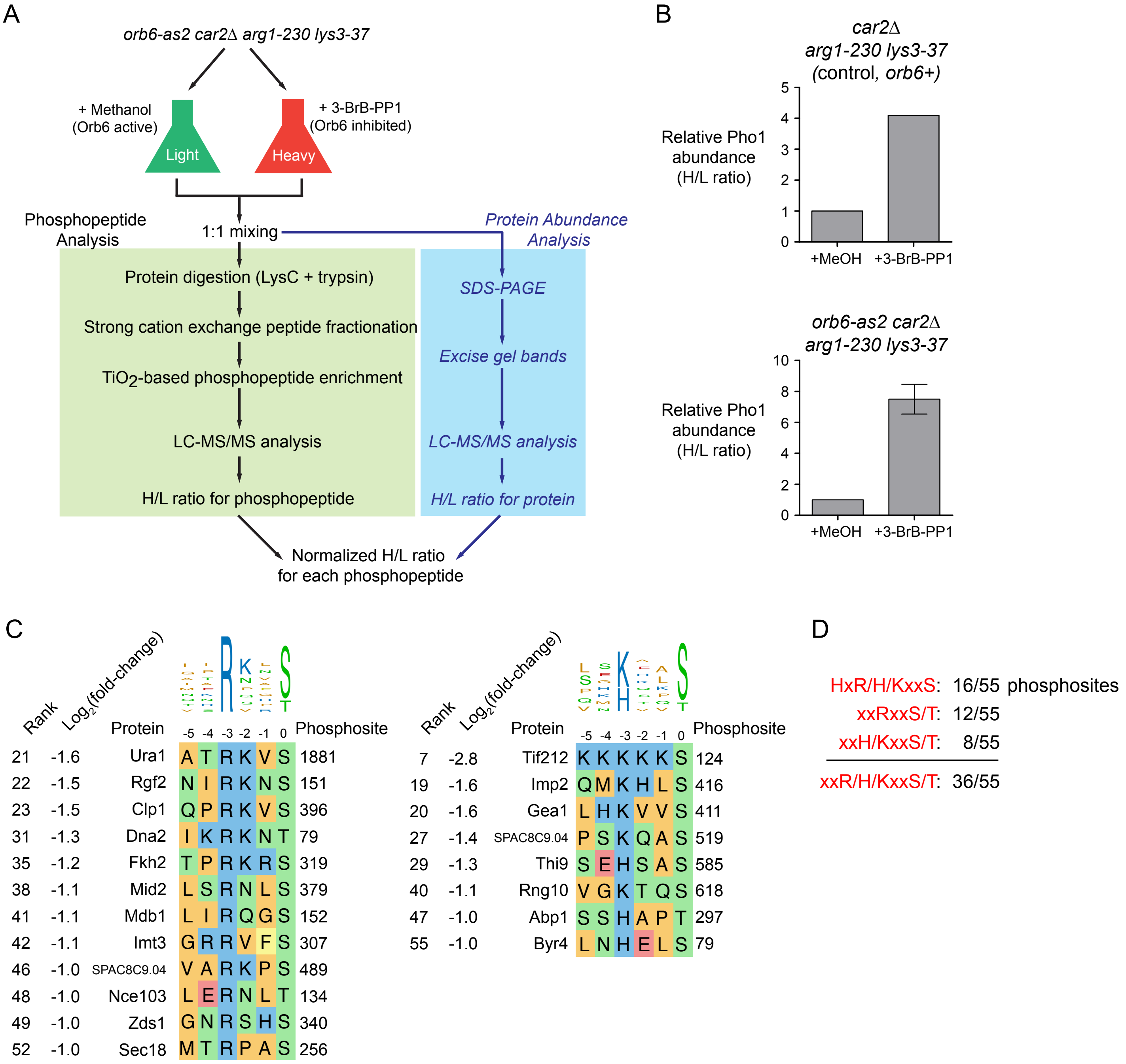

Figure S2. Quantitative global phosphoproteomics analysis after Orb6 inhibition. Related to Figure 4.

(A) Workflow for SILAC quantitative global phosphoproteomics analysis. Because relative abundance of individual proteins may change as a result of Orb6 inhibition, abundance of individual proteins before and after Orb6 inhibition is used to normalize H/L ratios of the relevant phosphopeptides.

(B) Relative abundance of acid phosphatase Pho1 after 3-BrB-PP1 addition to control wild-type (*orb6+*) cells and *orb6-as2* cells. Error bar indicates SD from three biological replicates. Relative abundances of other acid phosphatases SPBC4.06 and Pho4 were not significantly changed after 3-BrB-PP1 addition.

(C) Alignment of selected highest-confidence Orb6-dependent phosphosites containing basic residues R or H/K at -3 position. All phosphosites shown were quantified in all three replicate global phosphoproteomics experiments and showed a two-fold or greater mean decreased phosphorylation after Orb6 inhibition. Phosphosites are ranked by fold-decrease in phosphorylation (see Figure 4B and Table S2).

(D) Consensus summary of features of highest-confidence Orb6-dependent phosphosites from (C), together with sites with perfect match to NDR/LATS consensus HxR/H/KxxS/T (Figure 4B). 36 out of 55 sites include a basic residue at -3 position.

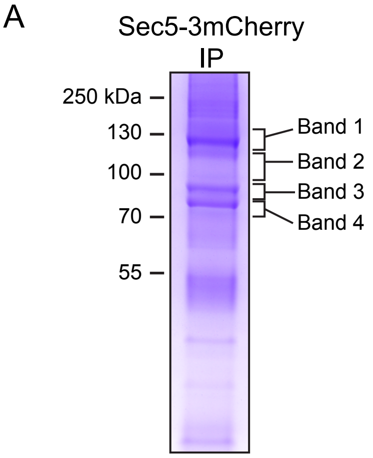

B

Protein	H/L Ratio	Peptide Counts
Sec5	1.05	51
Sec3	1.17	56
Sec8	0.99	89
Sec6	0.97	54
Sec10	0.79	14
Sec15	0.94	18
Exo84	0.83	10
Exo70	0.96	10

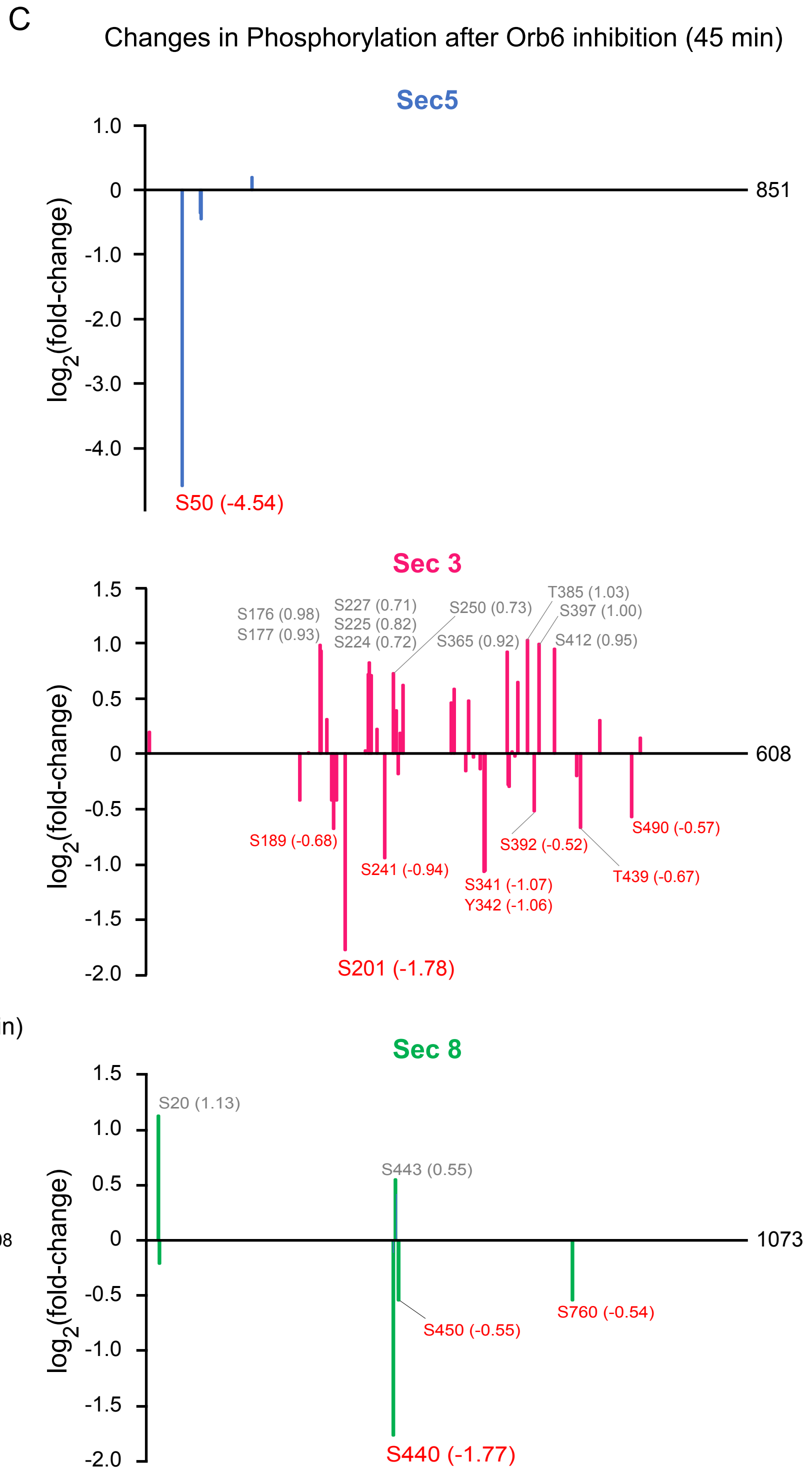

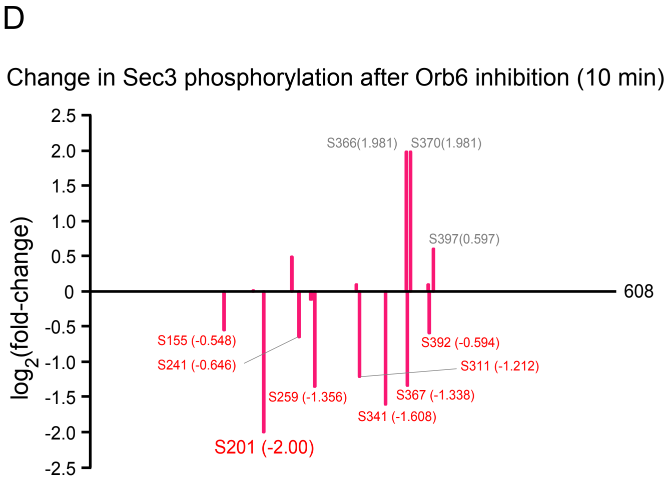

Figure S3. Additional confirmation of changes in phosphorylation of exocyst proteins after Orb6 inhibition. Related to Figure 4.
(A) SDS-PAGE showing immunoprecipitated Sec5-3mCherry and coimmunoprecipitated proteins, from a mixture of SILAC "heavy"-labelled Orb6-inhibited *orb6-as2* cells and "light"-labelled non-inhibited *orb6-as2* cells. Heavy-labelled cells were treated with 3-BrB-PP1, while light-labelled cells were treated with methanol. Indicated bands were excised and processed for tandem mass spectrometry.
(B) Relative abundances of exocyst proteins present in SILAC Sec5-3mCherry immunoprecipitate from (A) before and after Orb6 inhibition, based on SILAC heavy:light ratio (H/L ratio). All ratios are close to 1, suggesting that Orb6 inhibition does not grossly alter exocyst integrity. Note that H/L ratios here are specific to individual proteins immunoprecipitating with Sec5-3mCherry before vs. after Orb6 inhibition and are not indicating protein stoichiometry within the exocyst complex.
(C) Fold-changes in phosphorylation of individual phosphosites in Sec5, Sec3, and Sec8 after Orb6 inhibition for 45 minutes. X-axes show position of phosphosites in the respective proteins (Sec5 is 851 amino acids, Sec3 is 608 amino acids, and Sec8 is 1073 amino acids). Y-axes show $\log_2$(fold-change) in phosphorylation of relevant phosphosites after Orb6 inhibition. Among exocyst proteins, only Sec5, Sec3 and Sec8 showed significant changes in phosphorylation after Orb6 inhibition.
(D) Fold-changes in Sec3 phosphorylation after Orb6 inhibition for 10 minutes. In the 10-minute inhibition experiment, phosphorylation of Sec5-S50 and Sec8-440 was not detectable.

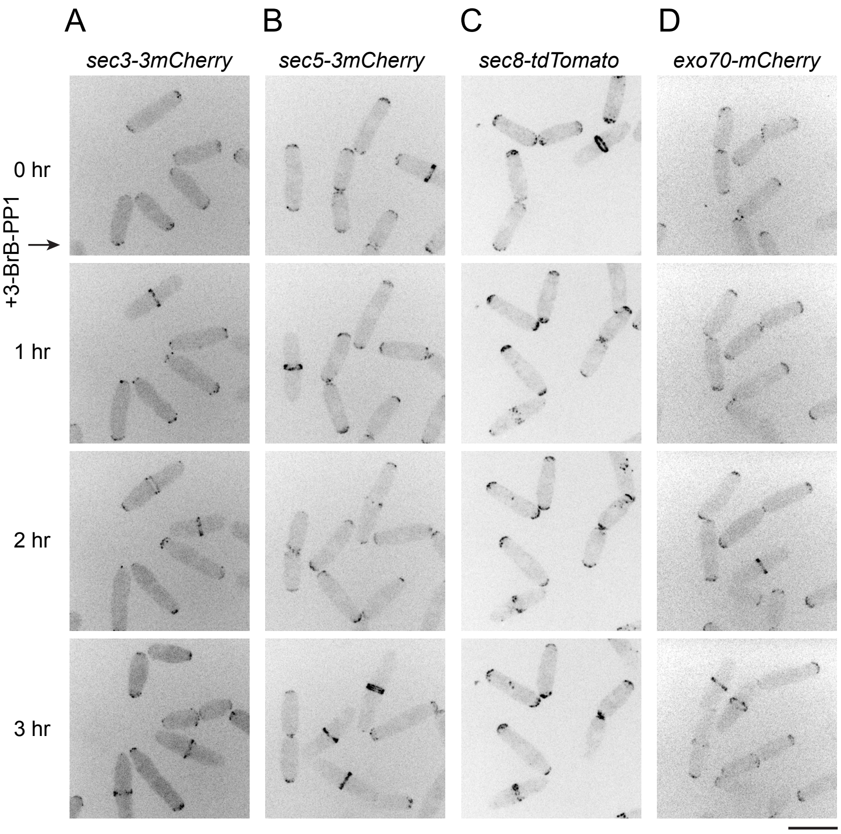

Figure S4. 3-BrB-PP1 treatment of wild-type cells does not affect exocyst localization. Related to Figure 6.
Localization of the indicated fluorescent-tagged exocyst proteins in wild-type (*orb6+*) cells, before 3-BrB-PP1 addition and at different times after 3-BrB-PP1 addition. In each column (A-D), the same fields of cells were imaged at all timepoints.
Bar, 10 μm. Imaging experiments for (A-D) were performed once.

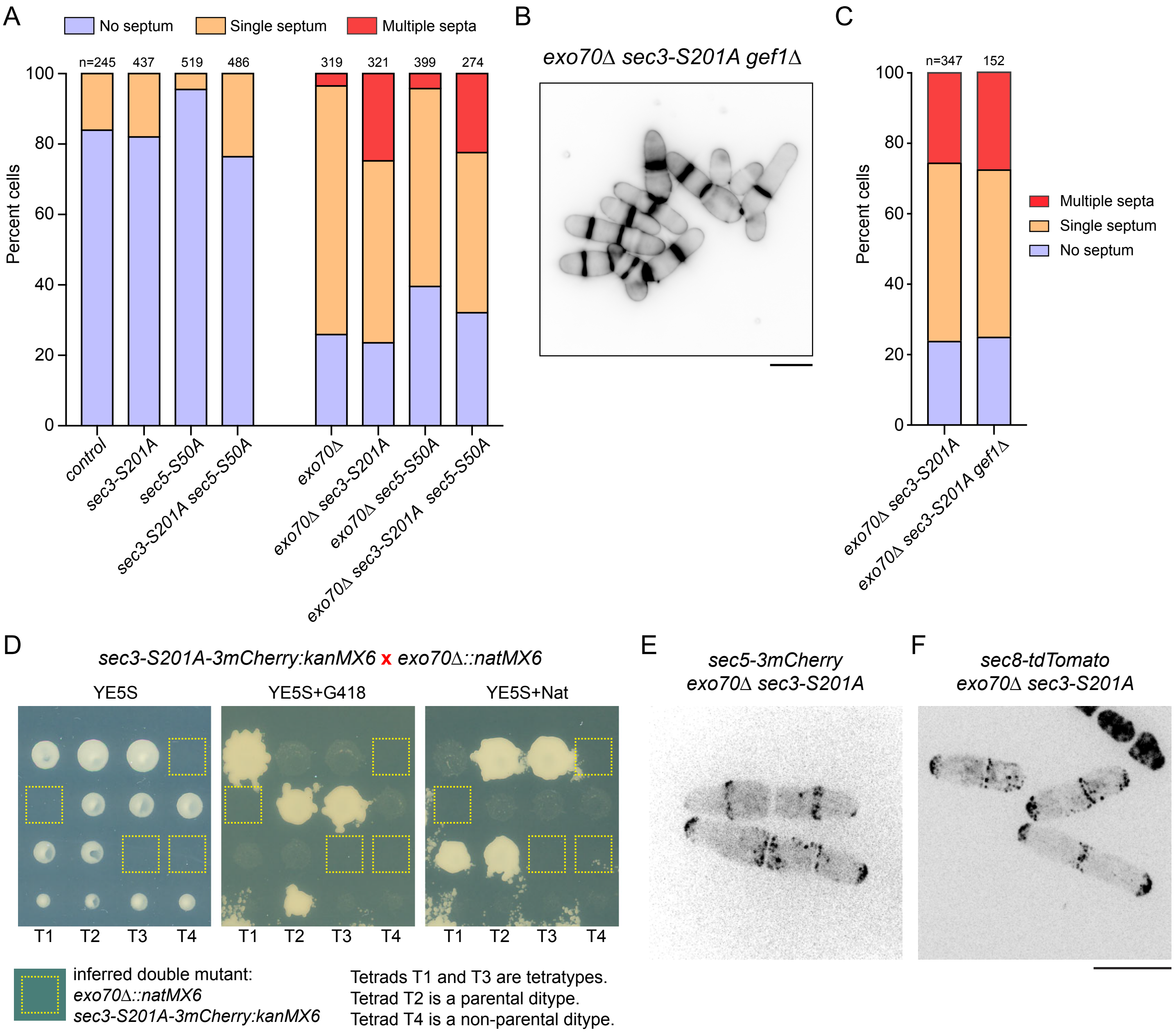

Figure S5. Further characterization of *sec3-S201A* and *sec5-S50A* phenotypes. Related to Figure 7.
(A) Septation index in cells of the indicated genotypes, grown at 36°C. See also Figure 7D
(B) Calcofluor staining of *exo70Δ sec3-S201A gef1Δ* cells.
(C) Septation index in cells of the indicated genotypes (25°C). Data in left-hand column are reproduced from Figure 7D.
(D) *sec3-S201A-3mCherry* is synthetically lethal with *exo70Δ*. As a result, Sec3-S201A-3mCherry localization in *exo70Δ* background cannot be investigated.
(E and F**)** Localization of Sec5-3mCherry (E) and Sec8-tdTomato (F) to cell tips and the septation zone in *exo70Δ sec3-S201A* backgrounds. In both strains, joined sister cells indicate cell-separation defects (see also Figure 7).
Bars, 10 µm. Imaging experiments and genetic cross for (A-F) were performed once.
